# Conjugation Pathway of Benzobisoxazoles in Polymer Donors Mediates the Charge Management and Enables Organic Solar Cells with Record Certified Efficiency

**DOI:** 10.1002/adma.202503702

**Published:** 2025-06-04

**Authors:** Miao Liu, Lunbi Wu, Yulong Hai, Yongmin Luo, Yao Li, Rouren Chen, Yue Ma, Tao Jia, Qingduan Li, Sha Liu, Ruijie Ma, Yue‐Peng Cai, Jiaying Wu, Gang Li, Shengjian Liu

**Affiliations:** ^1^ School of Chemistry Guangzhou Key Laboratory of Materials for Energy Conversion and Storage Key Laboratory of Electronic Chemicals for Integrated Circuit Packaging South China Normal University (SCNU) Guangzhou 510006 P. R. China; ^2^ School of Optoelectronic Engineering School of Mechanical Engineering Guangdong Polytechnic Normal University Guangzhou 510665 P. R. China; ^3^ Advanced Materials Thrust Function Hub The Hong Kong University of Science and Technology (Guangzhou) Nansha Guangzhou 511400 P. R. China; ^4^ Dongguan Key Laboratory of Interdisciplinary Science for Advanced Materials and Large‐Scale Scientific Facilities School of Physical Sciences Great Bay University Dongguan Guangdong 523000 P. R. China; ^5^ Department of Electrical and Electronic Engineering Research Institute for Smart Energy (RISE) Photonic Research Institute (PRI) The Hong Kong Polytechnic University Hong Kong P. R. China

**Keywords:** charge management, conjugation pathway isomerism, organic solar cell, polymer donor

## Abstract

Charge management plays a pivotal role in achieving high‐performance bulk heterojunction (BHJ) organic solar cells (OSCs). In this study, two efficient polymer donors are designed, P[4,8]BBO and P[2,6]BBO, by regulating the conjugation pathways of benzobisoxazoles (BBO) through 4,8‐ and 2,6‐linkages, respectively. Comparing to P[2,6]BBO, the isomer of conjugation pathway has been proved to enable P[4,8]BBO a shallower highest occupied molecular orbital (HOMO) energy level of −5.20 eV, significantly enhanced luminescence efficiency, and reduced aggregation property. These improvements lead to a dramatic increase in device efficiencies from 2.6% for P[2,6]BBO:eC9‐2Cl to 19.0% for P[4,8]BBO:eC9‐2Cl. The combined characterizations show that a better comprehensive charge management can be reached in P[4,8]BBO:eC9‐2Cl‐based OSCs, yielding a significantly higher short‐circuit current density (*J_SC_*) and fill factor (FF) parameters compared to P[2,6]BBO:eC9‐2Cl‐based ones. Furthermore, P[4,8]BBO demonstrates good applicability and can achieve an impressive efficiency of 19.4% in all‐polymer solar cells with a third‐party certified efficiency of 19.1%. This work highlights the critical role of conjugation pathway isomerism in mediating polymeric properties and advancing the development of high‐performance multifunctional photovoltaic materials.

## Graphical abstract

Herein, the conjugation pathway isomerism of the benzobisoxazole (BBO) building block is systematically compared, by which a certified efficiency of 19.1% is obtained in corresponding all‐polymer solar cells.

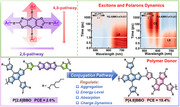

## 1 Introduction

As a new power generation technology, organic solar cells (OSCs) hold significant promise in photovoltaic market. This potential stems from advantages, including flexibility, lightweight, color adjustability, and compatibility with roll‐to‐roll printing technologies.^[^ ^1^, ^2^, ^3^, ^4^, ^5^, ^6^, ^7^ ^]^ Thanks to the innovation of non‐fullerene acceptors with strong light absorption in near‐infrared range (NIR, 700–1000 nm), high electron mobility, tunable energy level, and reduced non‐radiative energy loss, OSCs have reached power conversion efficiencies (PCEs) of up to 20%.^[^ ^8^, ^9^, ^10^, ^11^, ^12^, ^13^, ^14^ ^]^ Considering the strong light absorption of state‐of‐the‐art Y‐series acceptors in the near‐infrared region, developing efficient wide bandgap (WBG) polymer donors that exhibit good light absorption complementarity with Y‐series acceptors is of importance.^[^ ^15^, ^16^, ^17^, ^18^ ^]^ The alternating copolymerization of “weak acceptors” and “weak donors” is one of the most effective strategies for tailoring the optical band gap and absorption spectra, and reaching efficient polymer donors (PM6,^[^ ^19^ ^]^ D18,^[^ ^20^ ^]^ PTQ10,^[^ ^21^ ^]^ PBTz‐F,^[^ ^22^ ^]^ and their derivatives).

**Figure 1:**
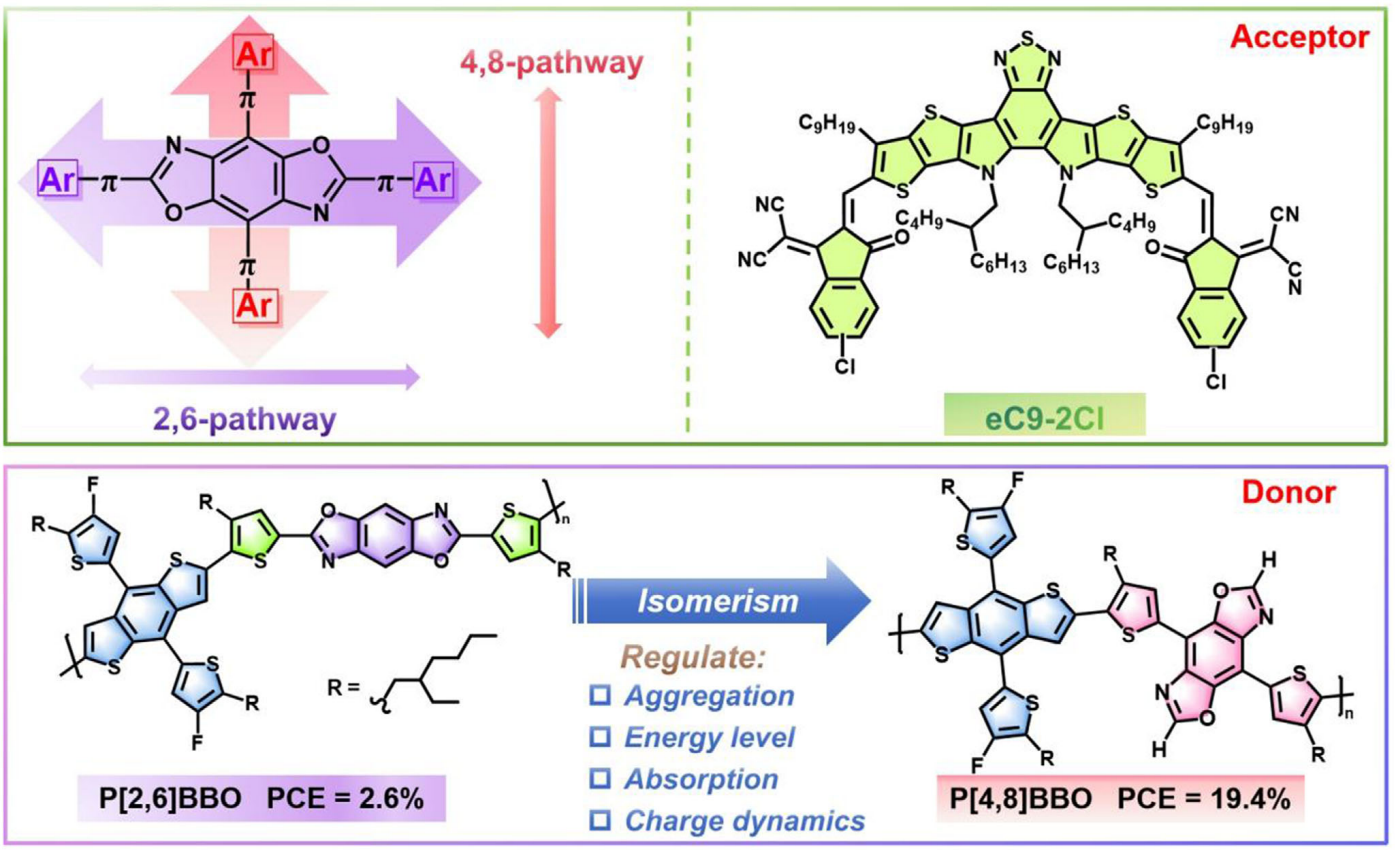
The two conjugation pathways of benzobisoxazole (BBO) and the chemical structures of eC9‐2Cl and polymeric isomers P[2,6]BBO and P[4,8]BBO.

Isomerism is a common phenomenon in organic and polymeric molecules and can be used to effectively tailor physicochemical properties of target materials.^[^ ^23^, ^24^, ^25^, ^26^, ^27^ ^]^ For example, Zhan et al. reported two acceptors, FNIC1 and FNIC2, with thiophene positional isomerism on the fused‐ring cores, demonstrating that optimizing the thiophene arrangement improves the absorption spectrum, electron mobility, affinity, and π–π stacking, leading to a higher power conversion efficiency (PCE).^[^ ^28^ ^]^ He et al. reported an electron‐accepting unit, TTDO, an isomer of building block for PM6 polymer, and the derived polymer donor named PBTT‐F can endow a red‐shifted absorption spectrum, more ordered packing compared to PM6.^[^ ^29^ ^]^ Recently, we demonstrated that the isomerism from 2,1,3‐benzothiadiazole to 1,2,3‐benzothiadiazole and naphtho [1,2‐c:5,6‐c']bis([1,2,5]thiadiazole) to naphtho [1,2‐c:5,6‐c']bis([1,2,3]thiadiazole) motifs can endow significantly distinctions of molecular configuration, energy level structure, luminous efficiency, and aggregation characteristic of target polymers and lead to complete difference in device performance.^[^ ^30^, ^31^ ^]^ Among various isomerism phenomena, conjugation pathway isomerism is a crucial yet underexplored aspect. In fact, the conjugation pathway influences the effective conjugation length, electron delocalization, dipole moment, and steric hindrance, thereby significantly impacting absorption spectra, molecular energy levels, aggregation behavior, and luminescence properties. For example, poly(N‐alkyl‐carbazole) with 2,7‐linkages exhibits a red‐shifted absorption spectrum, a narrower optical band gap, and a shallower highest occupied molecular orbital (HOMO) energy level compared to its 3,6‐linked isomer.^[^ ^32^, ^33^ ^]^ Benzobisazole (BBO) is known as a coplanar electron‐deficient aromatic heterocycle, featuring a central benzene ring fused with two oxazole rings.^[^ ^34^, ^35^, ^36^ ^]^ As shown in **Figure** **1**, the BBO structure typically exhibits 2,6‐ and 4,8‐conjugation pathways. However, most documented BBO‐based polymer donors have been π‐extended through the 2,6‐conjugation pathway, which generally leads to relatively poor photovoltaic performance.^[^ ^37^, ^38^ ^]^ In contrast, the transition from 2,6‐conjugation to 4,8‐conjugation may result in (i) increased steric hindrance due to the perpendicular orientation of the BBO building block and (ii) a shallower HOMO energy level caused by the exclusion of the BBO unit from the conjugated backbone. These merits potentially help to tailor energy levels, aggregation features, and also the BHJ morphology.

In this contribution, we focus on the isomerism of the conjugation pathway in benzobisoxazole (BBO) building blocks and report two novel polymers, P[2,6]BBO and P[4,8]BBO (Figure 1), which are π‐extended through 2,6‐ and 4,8‐conjugation pathways, respectively. The effect of this conjugation pathway distinction on the polymers' photoelectric properties and photovoltaic performance is systematically investigated. The transition from 2,6‐conjugation to 4,8‐conjugation enables precise energy level control, ensuring a sufficient driving force for exciton dissociation and balanced aggregation for an optimized blend film morphology, collectively contributing to superior charge management. Consequently, the P[4,8]BBO‐based OSCs achieved a significantly enhanced efficiency of 19.0%, with a short‐circuit current density (*J_SC_*) of 28.1 mA cm^−2^ and a fill factor (FF) of 77.5%, outperforming their P[2,6]BBO‐based counterparts. Moreover, the polymer donor P[4,8]BBO also demonstrated excellent performance in all‐polymer solar cells, achieving a high efficiency of 19.4%, with a third‐party verified efficiency of 19.1%. Our work demonstrates that benzobisoxazole (BBO) is a promising building block for designing efficient polymer donors, and that regulating conjugation pathways can effectively turn decay into magic in tailoring polymeric photoelectric properties.

## 2 Results and Discussion

### 2.1 Molecular Design, Synthesis and Characterization

**Figure 2:**
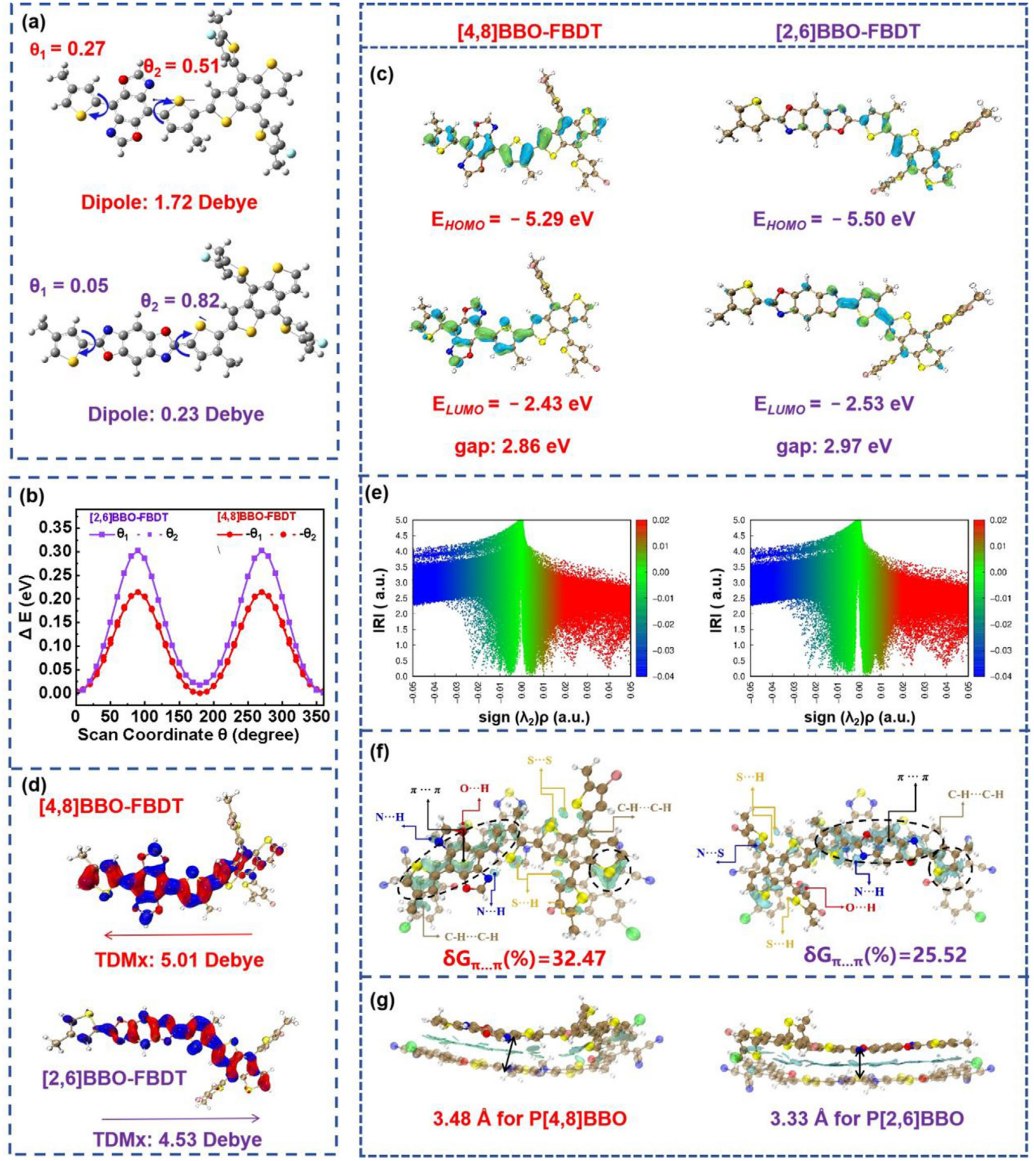
a) The optimal geometrical conformations and predicted dipole moment (µ), b) the potential energy surface (PES) scans of the torsion dihedral angles θ_1_ and θ_2_, c) the frontier molecular orbitals and energy level values, d) the hole and electron density distributions of S_0_ → S_1_ transition and transition dipole moments (TDMx) in x axis of backbone direction (blue and red represent the electron and hole distributions), e) Independent Gradient Model based on Hirshfeld (IGMH) and f) IRI isosurface map colored by sign (λ_2_)ρ of dimer made with donor and acceptor, g) the schematic diagram of molecular interactions between donors and acceptors.

To investigate the geometrical conformations and electronic structures, density functional theory (DFT) calculations were performed on two models,^[^ ^2^, ^6^ ^]^BBO‐FBDT and^[^ ^4^, ^8^ ^]^BBO‐FBDT, which represent the repeat units of P[2,6]BBO and P[4,8]BBO, respectively. First, the optimal conformations (**Figure** **2**a) reveal that both models exhibit small torsion dihedral angles (θ_1_: 0.05°–0.27° and θ_2_: 0.82°–0.51°) and nearly coplanar structures. The single‐crystal structure analyses further confirm the related model compounds demonstrate coplanar geometrical conformations with twist angles ranging from 3° to 6° between BBO unit and thiophene. Moreover, the^[^ ^4^, ^8^ ^]^BBO‐based model compound forms H‐aggregates with shorter π–π stacking distances of 3.507 Å and stronger intermolecular hydrogen bonding, which enhances charge transport between molecules (see Figure S30, Supporting Information). As shown in Figure 2b,^[^ ^4^, ^8^ ^]^BBO‐FBDT demonstrates a lower potential energy surface (PES) compared to^[^ ^2^, ^6^ ^]^BBO‐FBDT, indicating that the geometrical configuration of^[^ ^4^, ^8^ ^]^BBO‐FBDT is controllable. This suggests that the aggregation states of P[4,8]BBO can potentially be regulated by simply adjusting the solution processing parameters during device fabrication.^[^ ^39^ ^]^ Second, as shown in Figure 2c,^[^ ^4^, ^8^ ^]^BBO‐FBDT possesses a shallower HOMO energy level of −5.29 eV which provides a larger driving force for hole transfer from the acceptor to the donor. Third,^[^ ^4^, ^8^ ^]^BBO‐FBDT demonstrates a larger dipole moment (*µ*) of 1.72 Debye compared to^[^ ^2^, ^6^ ^]^BBO‐FBDT (0.23 Debye), facilitating intermolecular stacking and efficient charge transport.^[^ ^40^, ^41^, ^42^, ^43^ ^]^ Fourth, Figure 2e shows the hole and electron density distributions, indicating that both^[^ ^2^, ^6^ ^]^BBO‐FBDT and^[^ ^4^, ^8^ ^]^BBO‐FBDT exhibit a larger hole‐electron overlap (Sr‐index) value of 0.71–0.74. The transition dipole moments (TDMx) of^[^ ^2^, ^6^ ^]^BBO‐FBDT are directed from the acceptor BBO to the donor benzodithiophene (FBDT), while the opposite direction significantly inhibits the electron redistribution within the donor FBDT. In contrast,^[^ ^4^, ^8^ ^]^BBO‐FBDT shows large TDMx of 5.01 Debye, oriented from the donor FBDT to the acceptor BBO, which enhances the oscillator strength (*f*) to 1.58 and the light absorption coefficient to 0.655 × 10⁵ M⁻¹·cm⁻¹ (see Figure 2d; Tables S1 and S2 and Figure S2, Supporting Information). Lastly, the intermolecular interactions between the donor and acceptor were analyzed using the Interaction Region Indicator (IRI). As illustrated in Figure 2f,g, the red part represents the repulsion caused by the steric effect between donors and acceptors. The van der Waals interactions of the donor/acceptor molecules mainly originates from the π–π interactions of conjugated molecules.^[^ ^44^ ^]^ As predicted by the Independent Gradient Model based on Hirshfeld (IGMH),^[^ ^45^ ^]^ the^[^ ^4^, ^8^ ^]^BBO‐FBDT/eC9‐2Cl pair shows extremely strong π–π interactions and strong delocalized features of π‐electrons between the donor^[^ ^4^, ^8^ ^]^BBO‐FBDT and acceptor eC9‐2Cl molecules. While the interactions between^[^ ^2^, ^6^ ^]^BBO‐FBDT and eC9‐2Cl are relatively weaker. The parameter δG_π···π was introduced to describe the contribution of the π–π interaction. The^[^ ^4^, ^8^ ^]^BBO‐FBDT/eC9‐2Cl pair has a significantly larger δG_π···π value of 32.47% than that of^[^ ^2^, ^6^ ^]^BBO‐FBDT/eC9‐2Cl pair with δG_π···π of only 25.52%. The higher δG_π···π implies the^[^ ^4^, ^8^ ^]^BBO‐FBDT/eC9‐2Cl pair has better π–π stacking and stronger intermolecular interactions, which enhanced the charge transfer, separation, and generation between donor and acceptor.

**Scheme 1:**
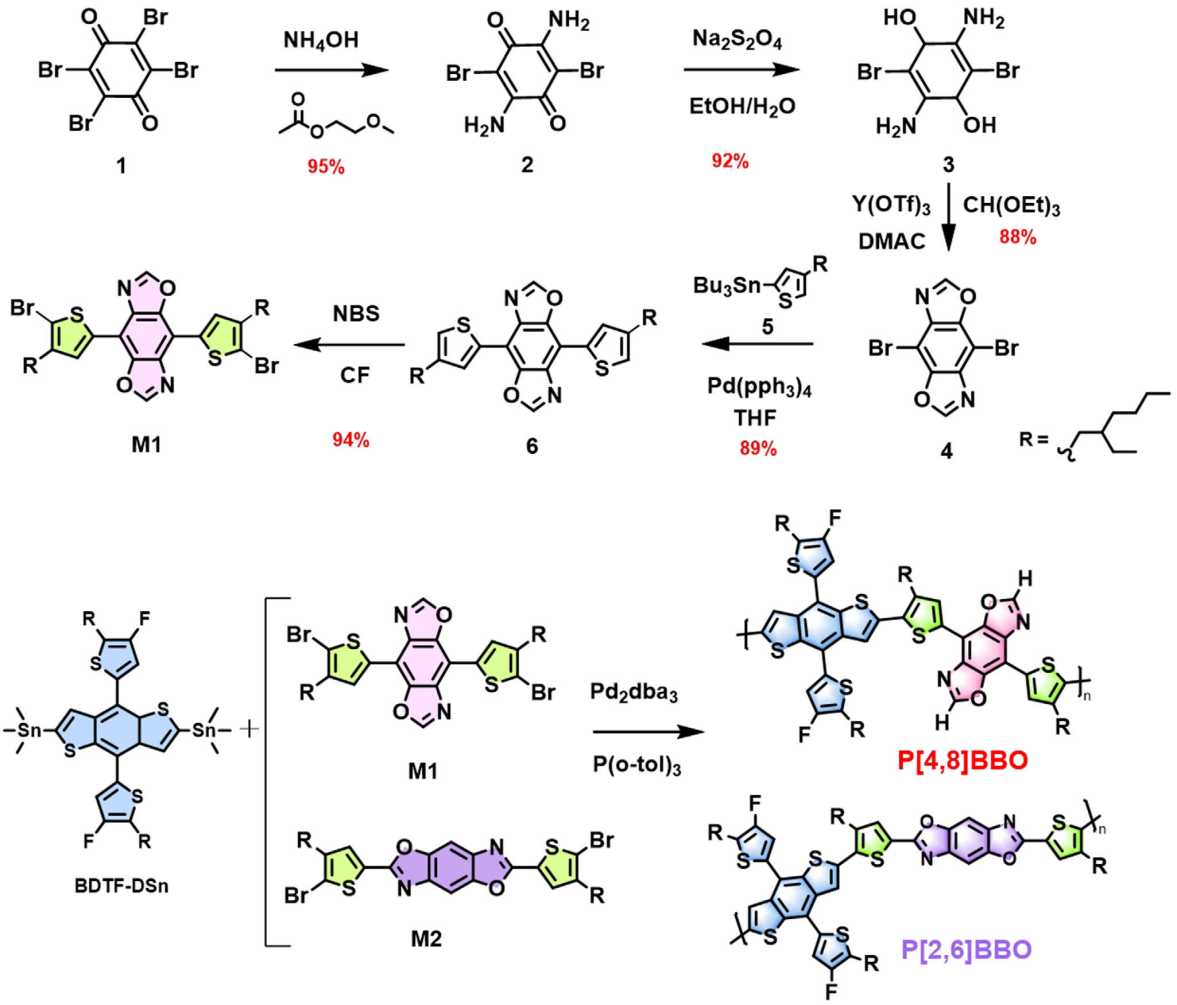
Synthetic routes for the key intermediate M1 and the two isomeric polymers, P[4,8]BBO and P[2,6]BBO.

Based on computational analysis, the conjugation pathway isomerism can precisely modulate the photoelectric properties. **Scheme** **1** outlines the synthetic routes for the key intermediate 4,8‐dibromobenzo[1,2‐d:4,5‐d']bis(oxazole) (**4**), the target polymer P[4,8]BBO, and the control polymer P[2,6]BBO. Briefly, the key intermediate (**4**) is synthesized from 2,3,5,6‐tetrabromocyclohexa‐2,5‐diene‐1,4‐dione 1) through an amine and bromine‐exchange reaction with ammonium hydroxide (NH₄OH), followed by quinone reduction using sodium dithionite (Na₂S₂O₄), and dehydrative condensation with triethyl orthoformate. Notably, these three reaction steps achieve a high overall yield of 77%, and all the key intermediates can be purified through recrystallization without the need for column chromatography, significantly reducing both material and labor costs for the synthesis. Afterwards, compound (**4**) undergoes a Stille coupling reaction with tributyl(4‐(2‐ethylhexyl)thiophen‐2‐yl)stannane (**5**) to yield intermediate (**6**), which is then brominated to produce the key monomer **M1**. The control monomer **M2** can be synthesized through a three‐step process, as previously reported.^[^ ^38^ ^]^ **M1** and **M2** were then polymerized via Stille coupling with the monomer **BDTF‐DSn** to produce P[4,8]BBO and P[2,6]BBO, respectively. The chemical structures has been well confirmed through ^1^H NMR, ^13^C NMR, high‐resolution mass spectrometry (HRMS), and high temperature gel permeation chromatography (GPC) characterizations (see Figures S3 and S4 and S10–S29 and Table S3, Supporting Information). P[4,8]BBO and P[2,6]BBO exhibit excellent solubility in common organic solvents, including chlorobenzene, chloroform, toluene, and *o*‐xylene, enabling their solution processability for the fabrication of OSCs.

### 2.2 Optical and Electronic Properties

**Figure 3:**
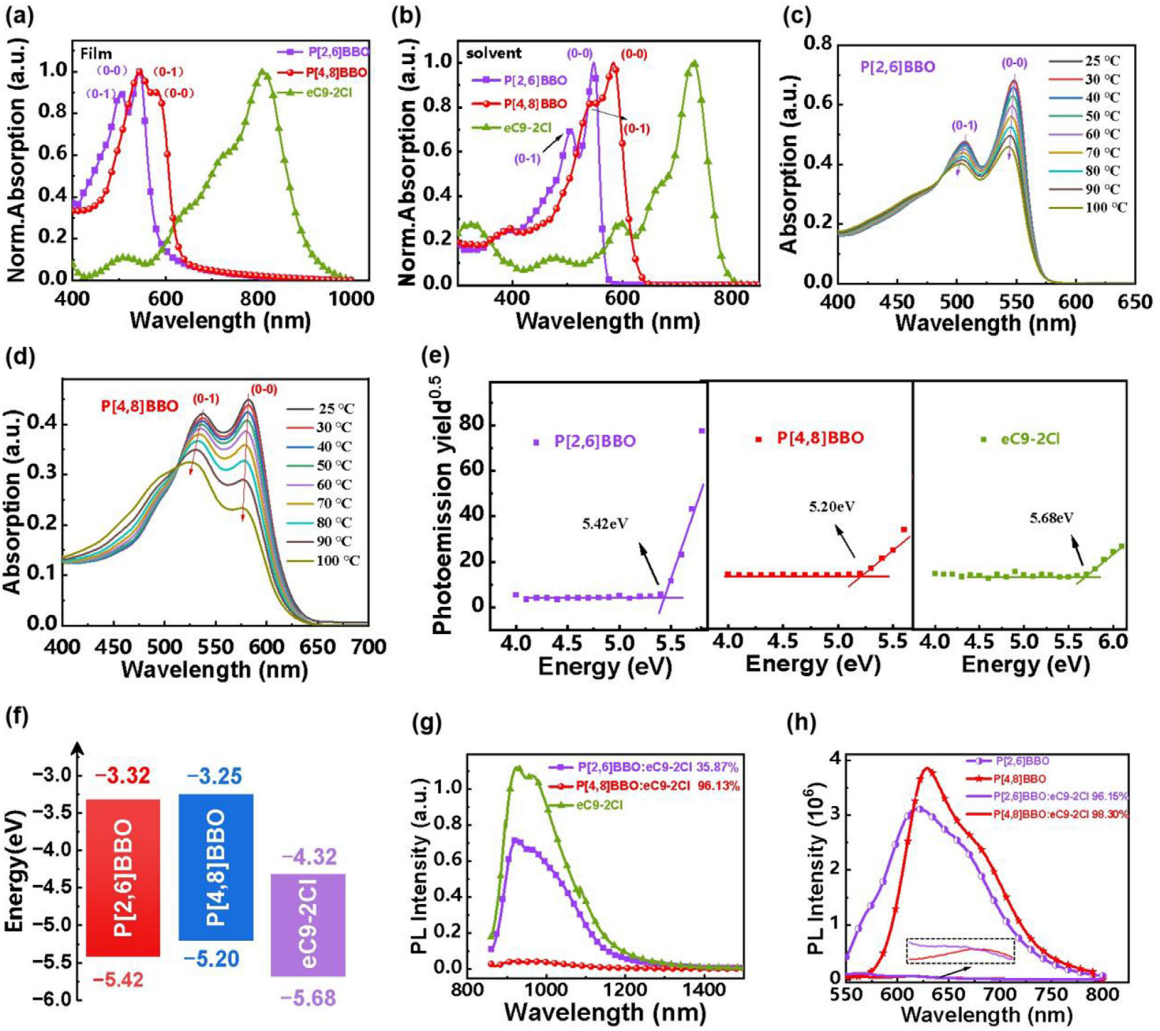
a,b) The UV–vis absorption spectra of P[4,8]BBO, P[2,6]BBO, and eC9‐2Cl in solid films and solutions; c,d) the temperature‐dependent UV–vis absorption spectra of P[4,8]BBO and P[2,6]BBO in solution; e,f) the PESA curves and energy levels for P[4,8]BBO, P[2,6]BBO, and eC9‐2Cl; the photoluminescence (PL) quenching of g) acceptor eC9‐2Cl in the presence of donors and h) donors in the presence of acceptor eC9‐2Cl as in optimized BHJ thin films.

**Figure** **3**a,b illustrates the UV–vis absorption spectra of the P[2, 6]BBO and P[4, 8]BBO donors and acceptor eC9‐2Cl, which exhibit complementary absorption across 400–1000 nm, enabling efficient light absorption in bulk heterojunction (BHJ) photoactive layers. Compared to P[2, 6]BBO, P[4, 8]BBO exhibits a bathochromic shift of ≈40 nm in both solution and films, accompanied by a reduction in the optical band gap (*E_g_^opt^*) from 2.10 to 1.95 eV (see **Table** **1**). Although both polymer donors exhibit 0–1 and 0−0 peaks, corresponding to the ICT effect and π–π* transition, their aggregation behaviors differ. P[4, 8]BBO shows lower I_0−0_/I_0−1_ ratios in both solution and film, comparedfilm, compare to P[2, 6]BBO, thereby indicating that P[4, 8]BBO forms controllable aggregates. Figure 3c,d presents the temperature‐dependent absorption spectra in solution. The I_0−0_/I_0−1_ ratio of P[2, 6]BBO remains high at 114.2% even at 100 °C, while that of P[4, 8]BBO significantly decreases from 106.7% at 25 °C to 71.9% at 100 °C (see Table S4, Supporting Information), confirming that the aggregation behavior is effectively modulated, potentially contributing to improved blend film morphology.^[^ ^46^ ^]^ The ionization potential (IP) values and energy level alignments, measured by photoelectron spectroscopy (PESA), are shown in Figure 3e,f. The control polymer donor P[2, 6]BBO exhibits a higher IP of 5.42 eV, corresponding to a deeper HOMO level and the energy offset for hole transfer between acceptor eC9‐2Cl and P[2, 6]BBO is only 0.26 eV, below the 0.3 eV typically required for efficient charge separation.^[^ ^47^, ^48^ ^]^ In contrast, P[4, 8]BBO exhibits a smaller IP of 5.20 eV, corresponding to a shallower HOMO level, which increases the HOMO energy offset between acceptor eC9‐2Cl and P[4, 8]BBO to 0.48 eV, potentially providing a stronger driving force for efficient hole transfer and charge generation.

**Table 1:** The molecular weight, optical and electronic properties of P[2,6]BBO, P[4,8]BBO, and eC9‐2Cl.

Polymer	M_n_ [kDa]	M_w_ [kDa]	*λ_max_^sol.^* [nm]	*λ_max_^film^* [nm]	*λ_onset_^film^* [nm]	*E_g_^opt^* ^a)^ [eV]	IP^b)^ [eV]
P[2,6]BBO	37.3	65.9	548	504/544	590	2.10	5.42
P[4,8]BBO	59.0	108.3	584	544/582	636	1.95	5.20
eC9‐2Cl	−	−	729	811	911	1.36	5.68

^a)^ $E_{g}^{opt}=1240/{\lambda_{\mathrm{onset}}}^{\mathrm{film}}$;

^b)^ IP determined by PESA.

Charge transfer between the donor and acceptor was further investigated through photoluminescence (PL) quenching experiments. As shown in Figure 3g,h, the high PL quenching efficiencies of P[2,6]BBO (≈96.15%) and P[4,8]BBO (≈98.30%) in the presence of eC9‐2Cl indicate efficient electron transfer from the donor domains to the acceptor domains. For hole transfer, the PL quenching efficiencies of eC9‐2Cl with P[2,6]BBO and P[4,8]BBO are 35.87% and 96.13%, respectively. The significantly higher quenching efficiency in the P[4,8]BBO:eC9‐2Cl system suggests more efficient hole transfer from eC9‐2Cl to P[4,8]BBO, consistent with the larger HOMO energy offset between P[4,8]BBO and eC9‐2Cl. These results highlight the critical role of the polymer donor's conjugation pathway in regulating energy levels and optimizing charge management.^[^ ^49^ ^]^

### 2.3 Photovoltaic Performance

**Figure 4:**
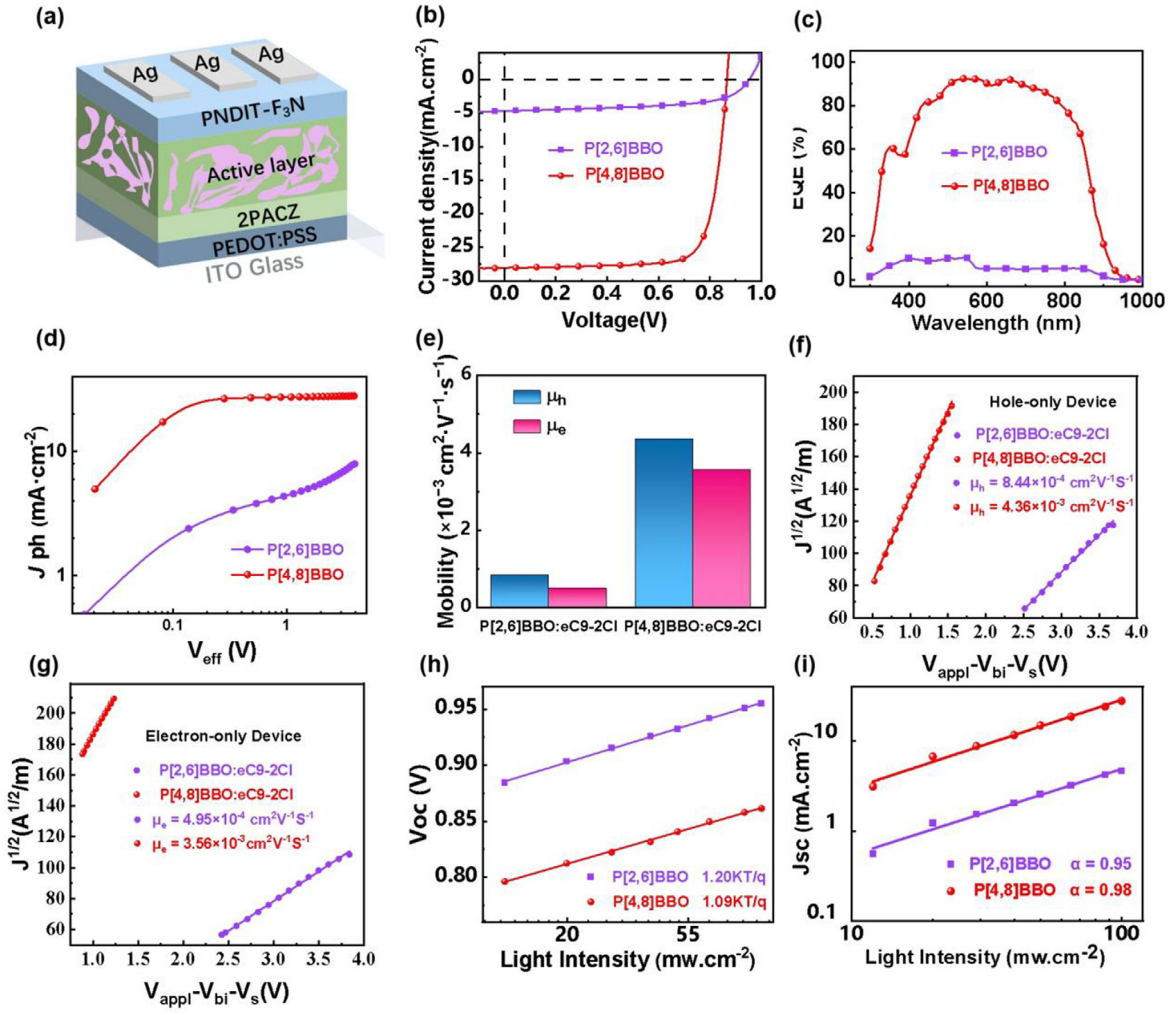
a) The device structure of binary OSCs, the b) *J–V* curves, c) EQE spectra, d) *J_ph_ vs. V_eff_* curves, e) hole and electron mobilities, f,g) dark current density versus voltage curves that were fitted by SCLC model, h,i) dependence of *V*_OC_ and *J*_SC_ as a function of incident light intensities (*P_light_*) for the P[2,6]BBO‐ and P[4,8]BBO‐based devices.

To investigate the impact of conjugation pathways on photovoltaic performance, P[4,8]BBO and P[2,6]BBO were employed as donors in OSCs (**Figure** **4**a), with eC9‐2Cl as the acceptor. The significantly different current density versus voltage (*J–V*) curves in Figure 4b demonstrate the profound influence of conjugation pathways on device performance. As summarized in **Table** **2**, P[2,6]BBO achieved a higher open‐circuit voltage (*V_OC_*) of 0.95 V due to its deeper HOMO level (−5.42 eV), but it exhibited a low short‐circuit current density (*J_SC_*) of 5.0 mA cm^−2^ and a poor fill factor (FF) of 55.2%, resulting in a very low power conversion efficiency (PCE) of 2.6%. Notably, previous studies have shown that BBO‐based polymer donors with a 2,6‐conjugation pathway typically achieve modest efficiencies ≈3% (Table S5, Supporting Information), which has limited the appeal of benzobisazole (BBO) as a candidate for designing high‐performance polymer donors.^[^ ^37^, ^50^, ^51^ ^]^ As shown in Table 2 and Figure 4b, the P[4,8]BBO‐based device achieved a slightly lower *V_OC_* of 0.87 V but exhibited significant improvements in *J_SC_* and FF parameters. Specifically, the *J_SC_* increased from 5.0 to 28.1 mA cm^−2^, and the FF improved from 55.2% to 77.5%, resulting in a remarkable PCE of 19.0%—approximately seven times higher than that of the P[2,6]BBO‐based device. Notably, this PCE value represents the highest efficiency reported for OSCs using BBO‐based polymer donors to date (Table S5, Supporting Information). These results demonstrate that benzobisazole (BBO) can be a promising building block for designing efficient polymer donors by ingenious structural design. The external quantum efficiency (EQE) spectra (Figure 4c) were measured to validate the *J_SC_* values. The P[2,6]BBO device shows a weak EQE response, with values below 10% across the 400–950 nm range, consistent with its low photoluminescence (PL) quenching efficiency and inefficient charge separation. In contrast, the P[4,8]BBO device achieves a significantly enhanced EQE response, exceeding 80% over the 500–800 nm range, indicating highly efficient photocurrent generation. The integrated photocurrents of 2.5 mA cm^−2^ for P[2,6]BBO and 27.2 mA cm^−2^ for P[4,8]BBO further confirm the superior performance of the P[4,8]BBO‐based device.

**Table 2:** The photovoltaic parameters of P[2,6]BBO‐ and P[4,8]BBO‐based binary OSCs.

Donor	*V_OC_* [V]	*J_SC_* [mA cm^−2^]	FF [%]	PCE [%]	*J_SC_^EQE^* [mA cm^−2^]
P[2,6]BBO	0.95	5.0	55.2	2.6 (2.4 ± 0.3)^a)^	2.5
P[4,8]BBO	0.87	28.1	77.5	19.0 (18.9 ± 0.1)^a)^	27.2

^a)^ the average values based on 16 independent devices.

### 2.4 Charge Generation, Transportation, and Recombination

Charge generation and collection were analyzed to understand the differences in FF, *J_SC_*, and efficiency. Figure 4d shows the photocurrent density (*J_ph_*) as a function of effective voltage (*V_eff_*). In the P[2,6]BBO device, *J_ph_* increases slowly with *V_eff_* and fails to saturate even at high *V_eff_*, indicating inefficient charge generation under built‐in electric field.^[^ ^52^ ^]^ In contrast, the P[4,8]BBO device shows a rapid increase in *J_ph_* with *V_eff_* (*V_eff_* < 0.2 V) and saturates when *V_eff_* > 0.2 V, with *J_ph_* becoming independent of *V_eff_*, suggesting efficient charge collection at the electrodes. The charge generation probabilities (*P(E,T)* = *J_ph_*/*J_sat_*) are 98.2% for P[4,8]BBO and 62.5% for P[2,6]BBO. The higher *P(E,T)* of P[4,8]BBO indicates more efficient charge generation and exciton dissociation, aligning with findings from ultrafast transient absorption studies (discussed later). Additionally, hole (*µ_h_*) and electron (*µ_e_*) mobilities were measured using space‐charge‐limited current (SCLC) method (Figure 4e–g). The P[4,8]BBO device exhibits *µ_h_* and *µ_e_* values of 4.36 × 10⁻^3^ and 3.56 × 10⁻^3^ cm^2^·V⁻¹·s⁻^1^, respectively, approximately five and seven times higher than those for the P[2,6]BBO devices. The *µ_h_*/*µ_e_* ratio is 1.71 for P[2,6]BBO and 1.22 for P[4,8]BBO, indicating more balanced charge transport in the P[4,8]BBO devices. Furthermore, *V_OC_* and *J_SC_* were measured under varying light intensities (*P_light_*). As shown in Figure 4h,i, the *n* value for the P[2,6]BBO device is 1.20, while for the P[4,8]BBO device, it is 1.09—closer to 1—indicating significantly reduced monomolecular and trap‐assisted recombination.^[^ ^53^ ^]^ The α values are 0.95 for P[2,6]BBO and 0.98 for P[4,8]BBO, demonstrating suppressed bimolecular recombination in the P[4,8]BBO device.^[^ ^54^ ^]^ These results highlight that regulating the conjugation pathway finely tunes charge behavior, leading to improved FF and higher *J_SC_*.

### 2.5 Morphological Characterization

**Figure 5:**
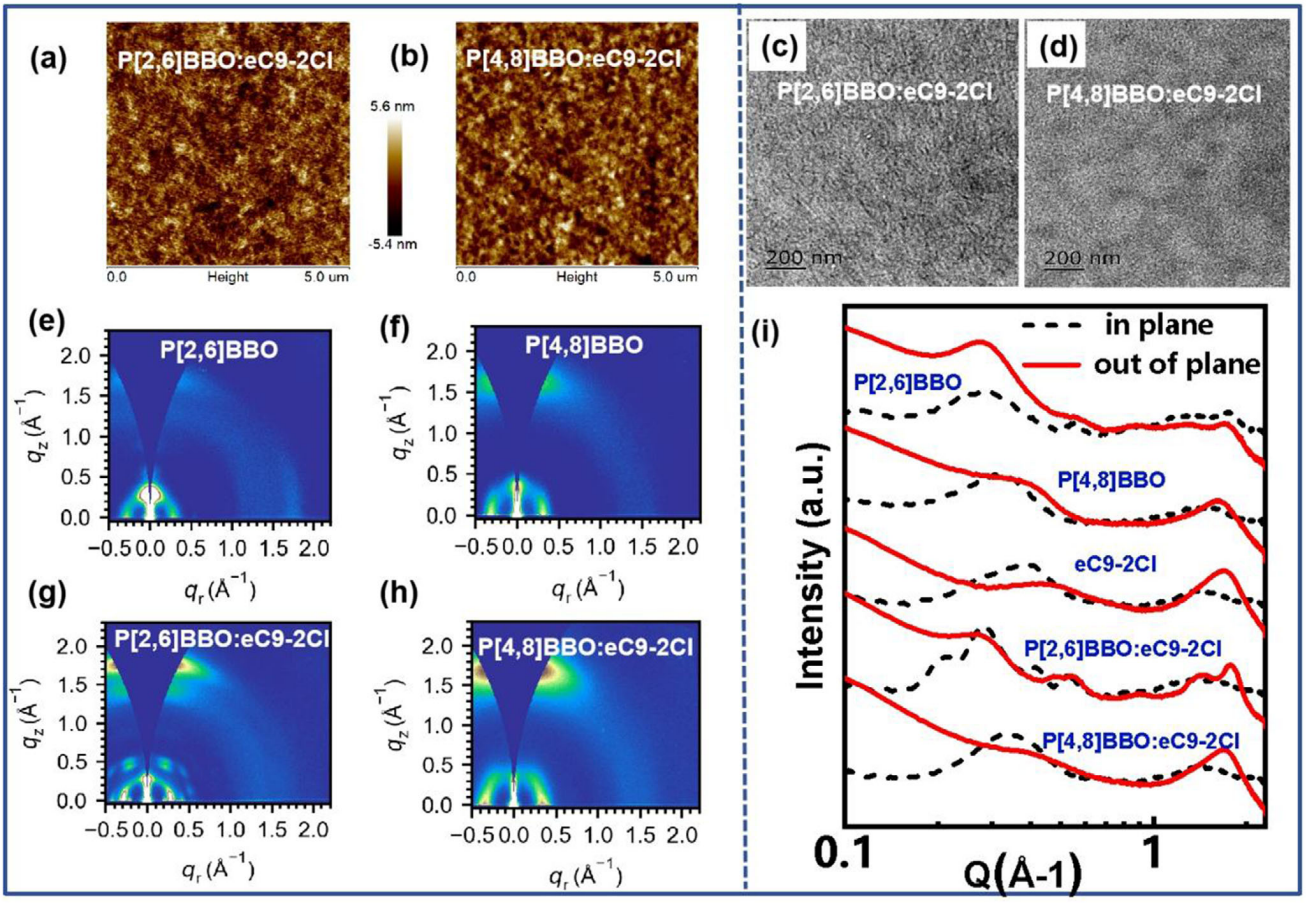
a,b) The AFM images and c,d) TEM images for the P[2,6]BBO:eC9‐2Cl and P[4,8]BBO:eC9‐2Cl BHJ films; the 2D‐GIWAXS patterns: e) P[2,6]BBO, f) P[4,8]BBO, g) P[2,6]BBO:eC9‐2Cl, and h) P[4,8]BBO:eC9‐2Cl; i) 1D‐GIWAXS line‐cut profiles of neat and BHJ films.

Atomic force microscopy (AFM), transmission electron microscopy (TEM), and grazing incidence wide‐angle X‐ray scattering (GIWAXS) were used to analyze the BHJ morphologies. As shown in **Figure** **5**a,b, the P[2,6]BBO‐ and P[4,8]BBO‐based BHJ films exhibit similar root‐mean‐square (RMS) roughness values of 1.20 nm and 1.42 nm, respectively. Figure 5c,d shows no significant differences in TEM images. Figure 5e–i presents the 2D GIWAXS patterns, out‐of‐plane (OOP) and in‐plane (IP) line‐cut profiles, with key diffraction parameters summarized in Tables S7–S10 (Supporting Information). Both P[2,6]BBO and P[4,8]BBO neat films display distinct (100) lamellar stacking peaks (q_r_ = 0.281–0.315 Å⁻¹) in the IP direction and (010) π–π stacking peaks (q_z_ = 1.597–1.674 Å⁻¹) in the OOP direction. P[2,6]BBO exhibits larger lamellar and π–π stacking crystal coherence lengths (CCLs) of 51.53 and 33.07 Å, respectively, while P[4,8]BBO shows a significantly reduced π–π stacking CCL of 17.95 Å. In blend films, both P[2,6]BBO:eC9‐2Cl and P[4,8]BBO:eC9‐2Cl display clear (100) lamellar stacking peaks (q_r_ = 0.282–0.344 Å⁻¹) in the IP direction and (010) π–π stacking peaks (q_z_ = 1.673–1.769 Å⁻¹) in the OOP direction, indicating strong face‐on orientation that facilitates efficient vertical charge transport.^[^ ^55^, ^56^ ^]^

### 2.6 Exciton and Charge Carrier Dynamics

**Figure 6:**
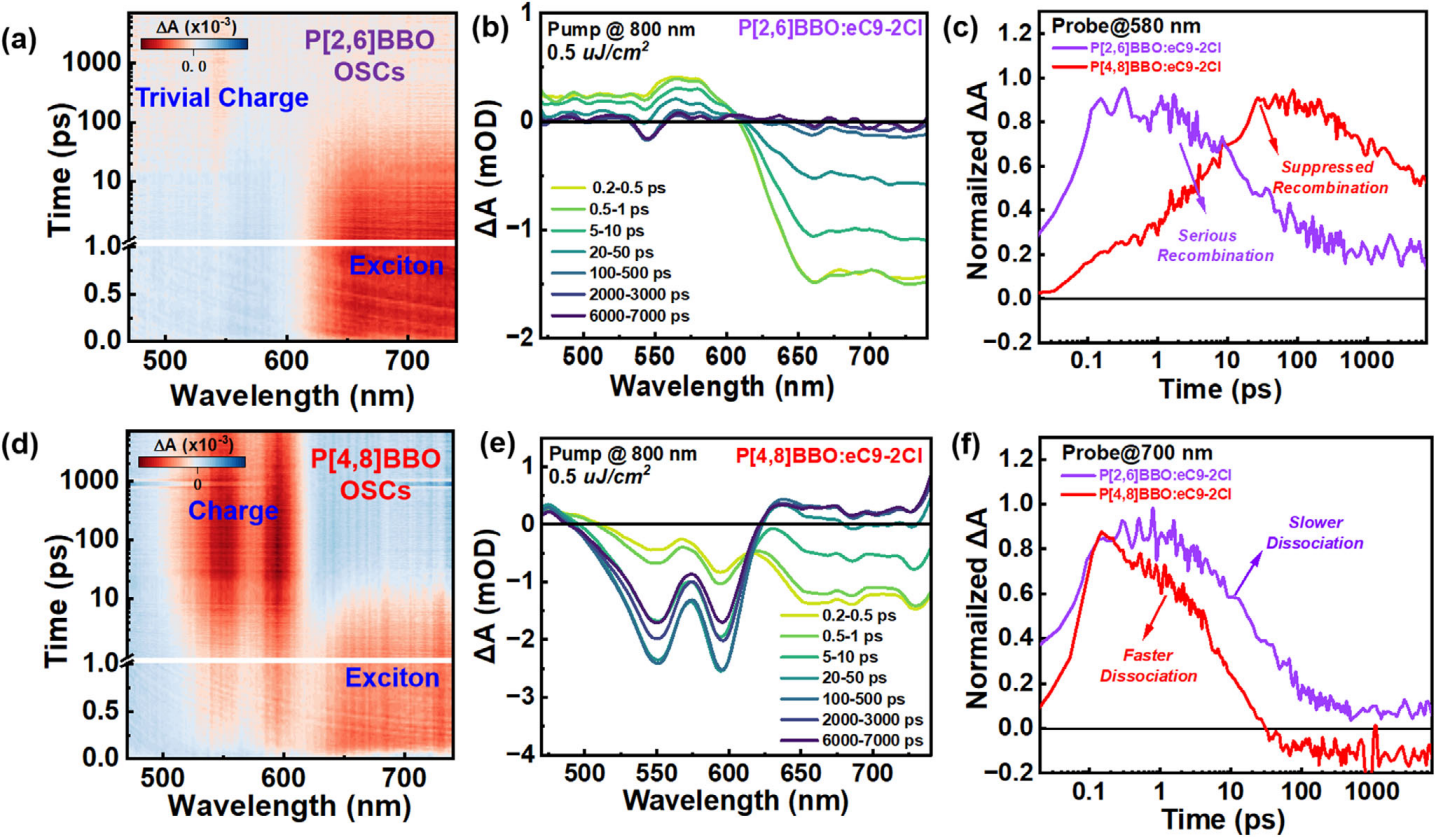
The femtosecond‐resolved a–d) TA signals, b–e) TA spectra, c–f) TA dynamic curves probed at 580 and 700 nm of P[2,6]BBO:eC9‐2Cl and P[4,8]BBO:eC9‐2Cl BHJ films excited at 800 nm.

To investigate charge behavior, exciton and polaron dynamics were analyzed using ultrafast transient absorption spectroscopy (TAS). Donor and acceptor neat films were excited with 550 nm and 800 nm lasers, and their TA spectra (Figure S8, Supporting Information) showed characteristic ground‐state bleach (GSB) peaks at 700 nm (eC9‐2Cl), 550 nm (P[2,6]BBO), and 550 nm (P[4,8]BBO), which were used to track exciton and polaron dynamics.^[^ ^57^, ^58^ ^]^ For P[2,6]BBO BHJ blends, an 800 nm laser excited the acceptor to generate eC9‐2Cl excitons and induce hole transfer. As shown in **Figure** **6**a,b,c,f: (i) the slow decay of the 700 nm peak in P[2,6]BBO:eC9‐2Cl indicates prolonged eC9‐2Cl exciton lifetime, (ii) the short‐lived signal at 580 nm, with lifetime below 100 ps, suggests a pure exciton signal and inefficient polaron generation, which aligns with their spectral characteristics of eC9‐2Cl, and (iii) the significant decay of the 580 nm peak indicates severe charge recombination. These factors corresponds to the lower *J_SC_* and FF found in P[2,6]BBO:eC9‐2Cl OSCs. Turning to the investigation of P[4,8]BBO:eC9‐2Cl BHJ film (Figure 6c–f): (i) the rapid decay of the 700 nm peak indicates efficient eC9‐2Cl exciton dissociation; (ii) the existence of long‐lived signal at 580 nm, peaking within 20–50 ps, confirms efficient P[4,8]BBO polaron generation due to a sufficient driving force of 0.48 eV; (iii) the long‐lived 580 nm signal suggests significantly suppressed recombination. These TAS results further highlight the critical role of the conjugation pathway in optimizing exciton dissociation, charge generation, and recombination, leading to enhanced *J_SC_* and FF.

### 2.7 All‐Polymer Solar Cells Characterizations

**Figure 7:**
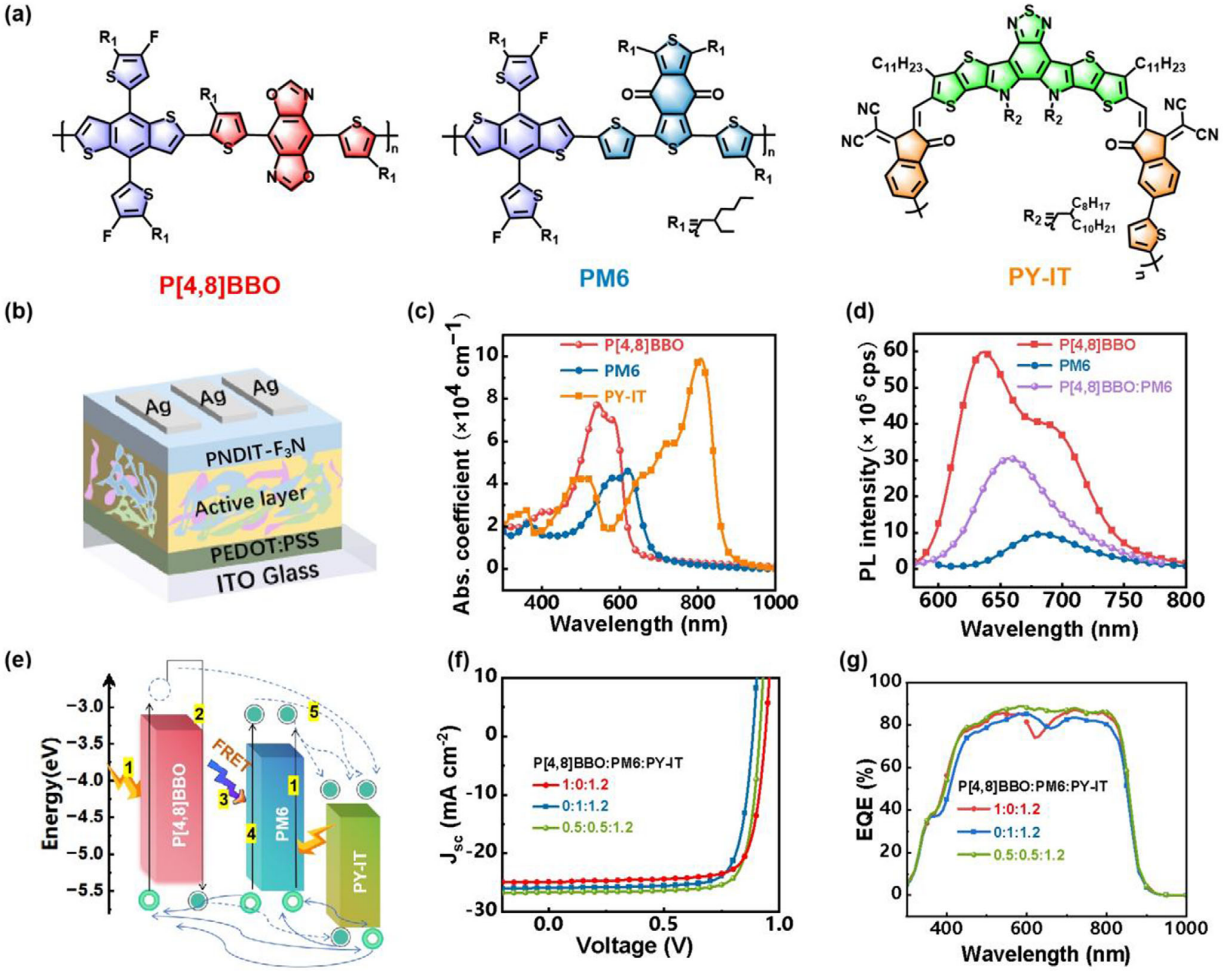
a) The chemical structure of P[4,8]BBO, PM6, PY‐IT, b) the device structure of all‐PSCs, c) UV–vis absorption spectra, d) photoluminescence (PL) spectra, e) energy level diagrams and possible pathways for Förster resonance energy transfer (FRET) and charge transfer, f) *J*–*V* curves and g) EQE spectra of binary and ternary cells based on P[4,8]BBO:PM6:PY‐IT system.

All‐polymer solar cells (all‐PSCs), featuring polymer donors and acceptors in the active layer, provide intrinsic long‐term stability and flexibility.^[^ ^59^, ^60^, ^61^ ^]^ Given its excellent performance, P[4,8]BBO is further examined and utilized as the donor in all‐polymer solar cells. As shown in **Figure** **7**: (i) P[4,8]BBO exhibits a large bandgap of 1.95 eV and strong absorption in the 450–620 nm range, complementing the absorption of the polymer donor PM6 (1.79 eV) and polymer acceptor PY‐IT (1.42 eV); (ii) it shows strong fluorescence emission with a high photoluminescence quantum yield (PLQY) of 8%, significantly surpassing the PM6 (≈2%); (iii) its photoluminescence in the 550–750 nm range well overlaps with the absorption spectra of PM6 (500–650 nm) and PY‐IT (650–850 nm), facilitating Förster resonance energy transfer (FRET) from P[4,8]BBO excitons to PM6 (Figure 7c);^[^ ^62^, ^63^ ^]^ (iv) its shallow energy level provides a larger driving force between P[4,8]BBO and PY‐IT, potentially enhancing exciton dissociation while suppressing geminate recombination at the donor/acceptor interface. These advantages make P[4,8]BBO a promising donor for efficient binary and ternary all‐PSCs.

Herein, P[4,8]BBO:PY‐IT and PM6:PY‐IT binary all‐PSCs were fabricated to evaluate the potential of P[4,8]BBO. As shown in Figure 7f and summarized in **Table** **3**, the benchmark PM6:PY‐IT device achieved a high V_OC_ of 0.94 V, FF of 77.9%, and J_SC_ of 24.9 mA cm^−2^, yielding a PCE of 18.2%. In comparison, P[4,8]BBO:PY‐IT exhibited a V_OC_ of 0.89 V, FF of 78.2%, and J_SC_ of 25.9 mA cm^−2^, resulting in a PCE of 17.9%, which is comparable to PM6‐based devices. As shown in Figure 7g, the P[4,8]BBO:PY‐IT blend achieves an EQE response over 80% across 450–900 nm but drops below 80% in the 600–660 nm range, whereas PM6:PY‐IT shows a higher EQE above 85% in that range. To address these inefficiencies, a ternary all‐PSC was fabricated by blending P[4,8]BBO and PM6 as donor components with PY‐IT as the acceptor. This strategy enhances energy level pairing and broadens the EQE response. With an optimal weight ratio of 0.5:0.5:1.2 for P[4,8]BBO:PM6:PY‐IT, the device efficiency improves from ≈18% in binary all‐PSCs to 19.4% in ternary all‐PSCs, driven by an increased FF of 79.7%, along with enhanced *J_SC_* (26.6 mA cm^−2^) and *V_OC_* (0.91 V). The increased *J_SC_* is directly confirmed by the EQE spectra in Figure 7g, where the ternary all‐PSCs show enhanced EQE across the entire spectrum mainly due to synergistic charge generation at the P[4,8]BBO/PY‐IT and PM6/PY‐IT interfaces. Notably, a third‐party certification verified an efficiency of 19.1% (Table 3, Figure S9, Supporting Information). To highlight its significance, Table S6 (Supporting Information) compares recent high‐PCE all‐PSCs certified by independent organizations. These findings further validate P[4,8]BBO as a promising polymer donor and demonstrate that tuning conjugation pathways is a powerful strategy for developing high‐performance polymers.

**Table 3:** Device performances of the optimal binary and ternary OSCs.

P[4,8]BBO:PM6:PY‐IT	*V*_OC_ [V]	*J*_SC_ [mA cm^−2^]	*J*_SC_^EQE^ [mA cm^−2^]	*FF* [%]	PCE [%]
1:0:1.2	0.89	25.9	25.1	78.2	17.9
0.5:0.5:1.2	0.91	26.6	25.9	79.7	19.4
0:1:1.2	0.94	24.9	24.2	77.9	18.2
Certified^a^ ^)^	0.89	27.0	−	79.5	19.1

^a)^ The ternary all‐PSCs certificated by independent organizations.

## 3 Conclusion

In summary, by tuning the conjugation pathway isomerism of benzobisoxazoles (BBO), we designed two polymer donors, P[2,6]BBO and P[4,8]BBO. We demonstrated that conjugation pathway isomerism effectively regulates multiple properties of the target polymers: (1) it optimizes the energy levels, providing a larger driving force for efficient charge transfer and exciton dissociation; (2) it regulates the backbone conformation to ensure appropriate aggregation and BHJ morphology; and (3) it enhances luminous efficiency, enabling efficient Förster resonance energy transfer (FRET). As the fruit of these merits, P[4,8]BBO‐based devices enable better charge management, with the derived binary OSCs achieving a high efficiency of 19.0%, significantly outperforming P[2,6]BBO‐based devices (2.6%). Furthermore, P[4,8]BBO performs well in binary all‐PSCs (≈18.0%) and improves ternary all‐PSCs to 19.4%, with a third‐party certified efficiency of 19.1%, ranking among the highest reported for all‐PSCs. This study underscores the potential of conjugation pathway engineering as an effective strategy for optimizing polymer properties, offering benefits not only for organic solar cells but also for broader organic electronic applications.

## Conflict of Interest

The authors declare no conflict of interest.

## Author Contributions

M.L., L.W., and Y.H. contributed equally to this work. S.L., T.J., R.M., and G.L. proposed the research and designed the experiments. M.L. and T.J. synthesized the polymers. L.W., R.C., and R.M. fabricated the BHJ OSCs. Y.H. conducted the simulated calculation. Y.L. and Y.L. carried out the experiments of GISAXS and TAS measurements and analyzed the TAS data. Y.M. and Q.L. help to analyze the data. S.L., Y.‐P. C., and G.L. provided the experiment condition. All authors commented on the manuscript.

## Supporting information



Supporting Infomation

## Data Availability

The data that support the findings of this study are available from the corresponding author upon reasonable request.
