# Supplementary material for: Conjugation Pathway of Benzobisoxazoles in Polymer Donors Mediates the Charge Management and Enables Organic Solar Cells with Record Certified Efficiency
Source: Adv Mater. 2025 Jun 4;37(33):2503702. doi: 10.1002/adma.202503702 (PMC12369686; doi:10.1002/adma.202503702)
Supplement: Supplementary file 1 — Supporting Infomation [file ADMA-37-2503702-s001.docx]

**Supporting Information**

**Conjugation Pathway of Benzobisoxazoles in Polymer Donors Mediates the Charge Management and Enables Organic Solar Cells with Record Certified Efficiency**

*Miao Liu,^a,†^ Lunbi Wu,^b,†^ Yulong Hai,^c,†^ Yongmin Luo,^c^ Yao Li,^c^ Rouren Chen,^a^ Yue Ma,^a^ Tao Jia,^b,^* Qingduan Li,^a^ Sha Liu,^d^ Ruijie Ma,^e,^* Yue-Peng Cai,^a^ Jiaying Wu,^c^ Gang Li,^e,^* and Shengjian Liu ^a,^**

*^a^*School of Chemistry, Guangzhou Key Laboratory of Materials for Energy Conversion and Storage, Key Laboratory of Electronic Chemicals for Integrated Circuit Packaging, South China Normal University (SCNU), Guangzhou 510006, P. R. China

*Email: [shengjian.liu@m.scnu.edu.cn](mailto:shengjian.liu@m.scnu.edu.cn) (Shengjian Liu)

*^b^*School of Optoelectronic Engineering, School of Mechanical Engineering, Guangdong Polytechnic Normal University, Guangzhou, 510665, P. R. China

*E-mail: [tjia@gpnu.edu.c](mailto:ruijie.ma@polyu.edu.hk)n (T. Jia).

*^c^*Advanced Materials Thrust, Function Hub, The Hong Kong University of Science and Technology (Guangzhou), Nansha 511400, Guangzhou, P. R. China

*^d^*Dongguan Key Laboratory of Interdisciplinary Science for Advanced Materials and Large-Scale Scientific Facilities, School of Physical Sciences, Great Bay University, Dongguan, Guangdong, 523000, P. R. China

*^e^*Department of Electrical and Electronic Engineering, Research Institute for Smart Energy (RISE), Photonic Research Institute (PRI), The Hong Kong Polytechnic University, Hong Kong, China

*E-mail: [ruijie.ma@polyu.edu.hk](mailto:ruijie.ma@polyu.edu.hk) (R. Ma); gang.w.li@polyu.edu.hk (G. Li)

**Content**

[1. Computational Analyses 4](#_Toc10558)

[1.1. DFT- Calculated Frontier Molecular Orbitals 5](#_Toc2533)

[1.2. DFT- Calculated UV-Vis Absorption Spectra and Excited State Characteristics 5](#_Toc2615)

[2. General Experimental Details 7](#_Toc9628)

[2.1. Materials 7](#_Toc13519)

[2.2. Synthetic Procedures 8](#_Toc32711)

[3. Gel Permeation Chromatography Measurements 13](#_Toc7961)

[4. UV−Vis Absorption Spectra 14](#_Toc17059)

[5. Photoelectron Spectroscopy in Air (PESA) Measurements 14](#_Toc3317)

[6. Photoluminescent (PL) spectra 14](#_Toc8923)

[7. OSCs Fabrication and Characterization 15](#_Toc1924)

[7.1. Device Fabrication. 15](#_Toc6058)

[7.2 Photovoltaic Performance of Benzobisazole based Donors. 16](#_Toc22856)

[8. Fabrication and Characterization of SCLC Devices 18](#_Toc30960)

[9. D Grazing Incidence Wide Angle X-ray Scattering (GIWAXS) 18](#_Toc31839)

[10. Atom Force Microscopy (AFM) Measurement 21](#_Toc23312)

[11.Transmission Electron Microscopy (TEM) Measurement 21](#_Toc4980)

[12. Ultra-fast Transient Absorption Spectroscopy Measurements 22](#_Toc32398)

[13. Certified Report of P[4,8]BBO:PM6:PY-IT-based all-PSCs. 23](#_Toc2910)

[14. Solution NMR and Mass Spectra 26](#_Toc6596)

[15. Crystal Growth and Single Crystal Determination 37](#_Toc12930)

[16. References 39](#_Toc15471)

# 1. Computational Analyses

Gaussian 16 (Revision C.02) code ^[1]^ was used to perform density functional theory (DFT) calculations at the non-empirically tuned B3LYP-D3(BJ)/TZVP ^[2-4]^ level of theory. The P[4,8]BBO and P[2,6]BBO were replaced as [2,6]BBO-FBDT and [4,8]BBO-FBDT monomer with methyl groups to reduce the computational cost. For the potential energy surface (PES) modeling, the dihedral angle between the thiophene and adjacent building blocks were scanned at an interval of 10° between the 0° and 360° conformations. In the calculation of highest occupied molecular orbital (HOMO) and lowest unoccupied molecular orbital (LUMO) energy levels, the single point energy was calculated under B3LYP-D3(BJ)/Def2TZVP ^[2-4]^ level for high precision calculations. We considered a total of 50 excited states from S_1_ to S_50_ at the theoretical level of *ω*B97X-D/Def2tzvp and calculated the electron-hole distribution and transition dipole moment (TDM) from S_0_ to S_1._ The wavefunction software Multiwfn ^[5]^ and VMD ^[6]^ were used for analyzing potential energy surface (PES) and electron-hole overlapping (Sr index). The larger the Sr index, the greater the overlap of holes and electrons; the smaller the value, the more significant is the separation of holes and electrons. The intensity corresponding to the jump from the ground state to the excited state is embodied in the UV-Vis spectrum (proportional to the integral area of the absorption peak) corresponds to the vibronic intensity. The square of the mode of the jump dipole moment is equal to the sum of the X, Y, and Z parts of the contribution.

## 1.1. DFT- Calculated Frontier Molecular Orbitals


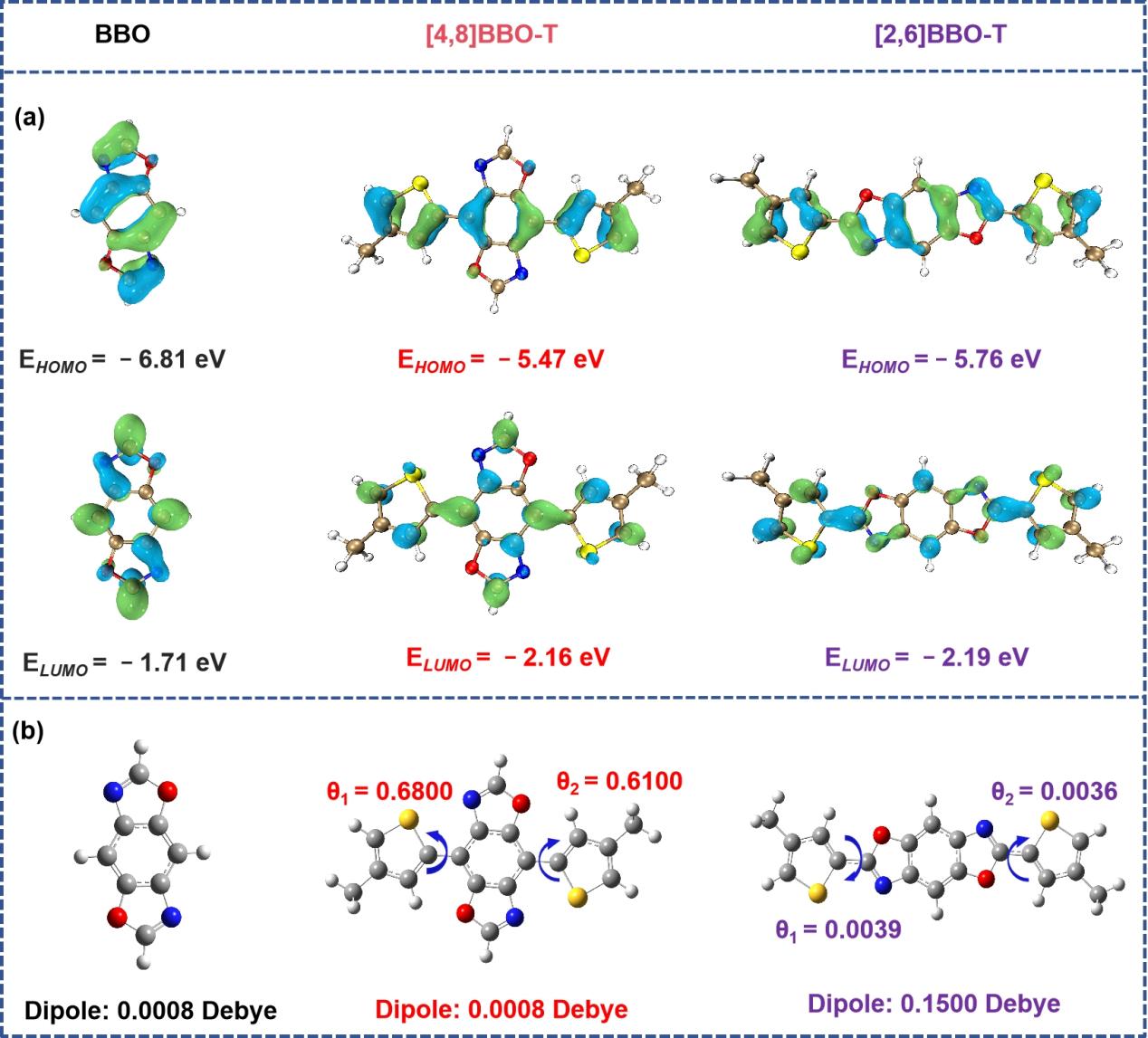


**Figure S1.** (a) The computed frontier molecular orbital profiles of BBO, [4,8]BBO-T and [2,6]BBO-T; (b) The optimal geometrical conformations of BBO, [4,8]BBO-T and [2,6]BBO-T.

## 1.2. DFT- Calculated UV-Vis Absorption Spectra and Excited State Characteristics

As provided in Figure S2, the black curves are the DTF-predicted UV-Vis spectra, and the red and blue curves represent the contribution of electron redistribution in the donor unit and acceptor unit to the absorption, respectively. The red dashed line and the blue dashed line represent the contribution of electron transfer from the donor unit to the acceptor unit (red dashed line) and from the acceptor unit to the donor unit (blue dashed line) to the absorption. As shown in Figure S2, it can be seen intuitively that for [4,8]BBO-FBDT, the absorption peaks at 230 nm and 380 nm are mainly caused by the redistribution of electrons within the donor unit. While for [2,6]BBO-FBDT, this trend is significantly weakened, and electron redistribution and intramolecular charge transfer jointly act on the absorption peak at 400 nm. Both intramolecular electron transfers make important contributions to the absorption, but [2,6]BBO-FBDT has a slightly higher absorption ratio, a total of 50%, while for P[4,8]BBO, it is 43.7%. However, for the contribution of electron redistribution within the donor unit, [4,8]BBO-FBDT is significantly stronger than that of [2,6]BBO-FBDT, possibly due to the large difference in transition dipole moments (TDM) in the x direction. The TDM value for [4,8]BBO-FBDT is 5.01, with the direction from D to A. While for [2,6]BBO-FBDT, due to the orientation of the acceptor unit, the TDM direction is from A to D, and the opposite direction significantly inhibits the electron redistribution of the D unit, weakening the absorption of [2,6]BBO-FBDT at 230 nm and 400 nm.


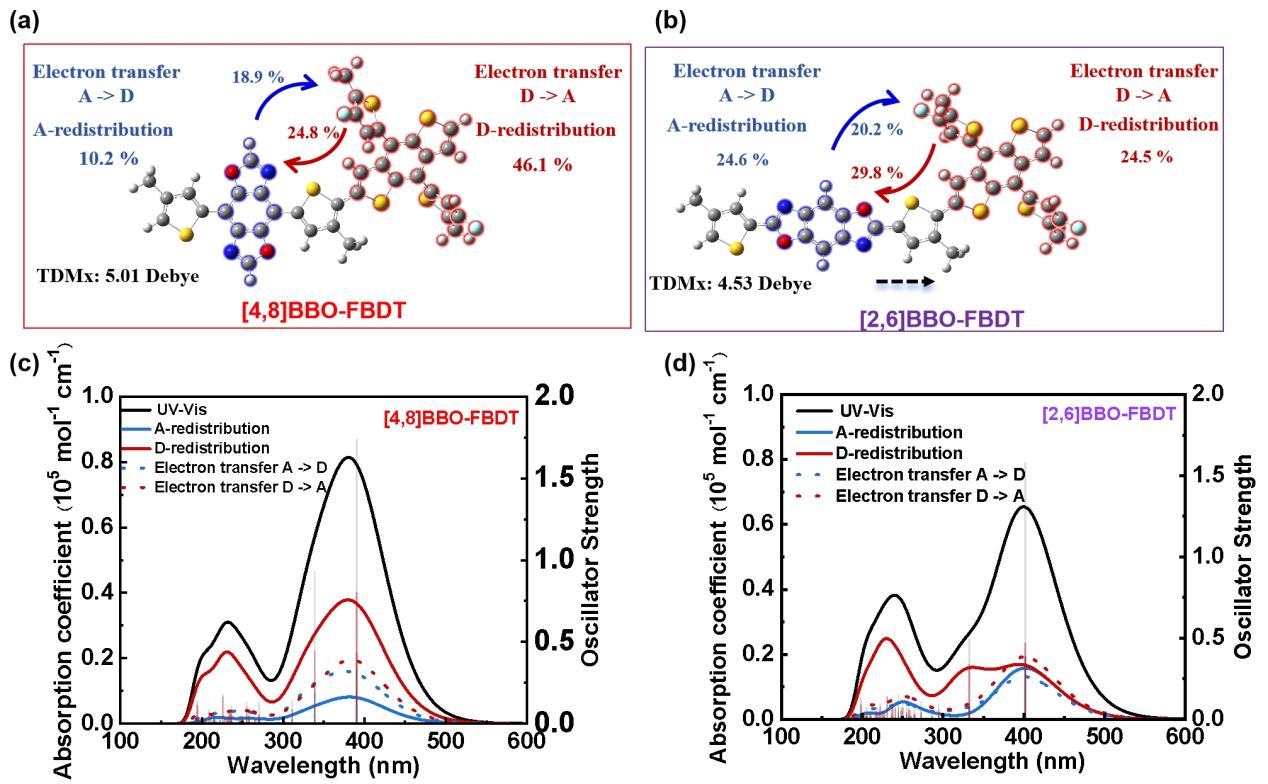


**Figure S2.** (a, b) The hole and electron density distributions, (c, d) the simulated UV-vis absorption spectra and oscillator strength (*f*) of [2,6]BBO-FBDT and [4,8]BBO-FBDT monomer.

**Table S1**. The predicted transition dipole moments (TDM) in x, y, z direction, Sr-index, oscillator strength (*f*) of [4,8]BBO-FBDT and [2,6]BBO-FBDT monomer.

| Model | TDM_x_  [a.u.] | TDM_y_  [a.u.] | TDM_z_  [a.u.] | Sr  [a.u.] | *f*  strength |
| --- | --- | --- | --- | --- | --- |
| [4,8]BBO-FBDT | 5.01 | 0.30 | 0.41 | 0.74 | 1.73 |
| [2,6]BBO-FBDT | 4.53 | 1.63 | 0.06 | 0.71 | 1.57 |

**Table S2.** The predicted HOMO and LUMO energy levels, dipole moment (*µ*), Sr-index, and $\delta G\_\pi\cdot\cdot\cdot\pi$.

| Model | *E*_HOMO_  [ev] | *E*_LUMO_  [ev] | *µ*  [Debye] | Sr  [a.u.] | $G\_\pi\cdot\cdot\cdot\pi$ |
| --- | --- | --- | --- | --- | --- |
| [4,8]BBO-FBDT | −5.29 | −2.43 | 1.72 | 0.74 | 32.47% |
| [2,6]BBO-FBDT | −5.50 | −2.53 | 0.23 | 0.71 | 25.52% |

# 2. General Experimental Details

## 2.1. Materials

2,3,5,6-tetrabromocyclohexa-2,5-diene-1,4-dione (1), tributyl(4-(2-ethylhexyl)thiophen-2-yl)stannane (5), (4,8-bis(5-(2-ethylhexyl)-4-fluorothiophen-2-yl)benzo[1,2-b:4,5-b']dithiophene-2,6-diyl)bis(trimethylstannane) (BDTF-DSn), PNDIT-F_3_N, PM6, eC9-2Cl and PY-IT were purchased from Solarmer Energy, Inc. Other chemicals and solvents were purchased from commercial sources (Sigma Aldrich, Acros, Stream, or Alfa Aesar) and used as received.

## 2.2. Synthetic Procedures


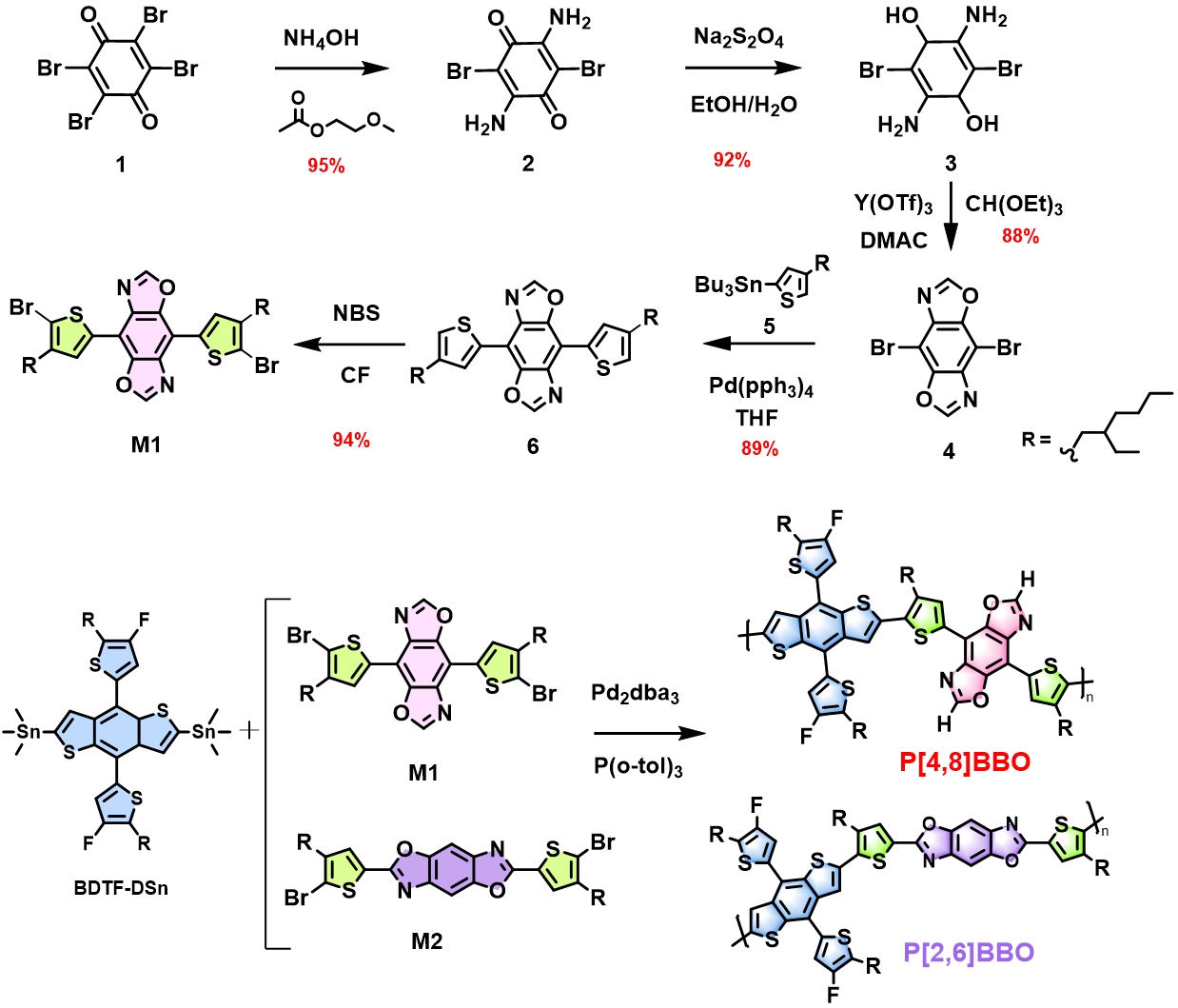


**Scheme S1**. The syntheses of monomer **M1**.

**2,5-Diamino-3,6-dibromocyclohexa-2,5-diene-1,4-dione (2):** To a stirred solution mixture of 2,3,5,6-tetrabromocyclohexa-2,5-diene-1,4-dione (**1**) (42.91 g, 100.0 mmol) in 170 mL 2-methoxyethyl acetate at 60 ^o^C, 70 mL ammonium hydroxide (27%) (300.0 mmol) was added dropwise over 0.5 hour under air atmosphere. The mixture was then vigorously stirred at 80 ^o^C for another 3 hours. After cooling to room temperature, the reaction mixture was filtered and the precipitate was collected and washed with large portions of distilled water and then dichloromethane. 2,5-Diamino-3,6-dibromocyclohexa-2,5-diene-1,4-dione (**2**) was quantitatively obtained as red powder, which was immediately used for the next step reaction (28.10 g), yield: 95%.

**2,5-Diamino-3,6-dibromobenzene-1,4-diol (3):** To a solution mixture of **2** (3.26 g, 110.0 mmol) in 40 mL ethanol:water mixed solvent (*v*:*v*, 5:1), Na_2_S_2_O_4_ (4.78 g, 27.0 mmol) dissolved in 150 mL water was added dropwise over 0.5 hour under argon atmosphere. The mixture was then stirred at 55 ^o^C for another 1 hour. The resulting mixture was cooled to room temperature. The precipitate was collected by filtration and the filter cake was washed by water and then cold methanol. The 2,5-diamino-3,6-dibromobenzene-1,4-diol was obtained as brown powder (3.04 g), yield: 92%. Due to the limited solubility and air sensitivity, **3** was immediately used for the next step reaction.

**4,8-Dibromobenzo[1,2-d:4,5-d']bis(oxazole) (4):** Y(OTf)_3_ (13.20 g, 40.0 mmol), 32 mL dimethylacetamide and triethoxymethane (20.00 g, 135.0 mmol) were added into a two-neck flask under argon atmosphere. The reaction mixture was allowed to be warmed to 60 ^o^C. The **3** (13.20 g, 44.0 mmol) was then added into the reaction mixture portion wise over 0.5 hours under argon atmosphere. The reaction was stirred at 60 °C for another 3 hours. After cooling to room temperature, the reaction mixture was filtered and the precipitate was collected and washed with distilled water and then cold methanol to directly obtain pure **4** as white powder (11.97 g), yield: 88%. The product was confirmed by ^1^H NMR spectroscopic analysis. ^1^H NMR (600 MHz, CDCl_3_) δ 8.30 (s, 2H). MALDI-TOF (m/z): [M]^+^ calcd. For C_8_H_2_Br_2_N_2_O_2_, 317.9240; found, 318.8537.

**4,8-Bis(4-(2-ethylhexyl)thiophen-2-yl)benzo[1,2-d:4,5-d']bis(oxazole) (6)****:** The **4** (1.00 g, 3.0 mmol), tributyl(4-(2-ethylhexyl)thiophen-2-yl) stannane (**5**) (3.58 g, 10.0 mmol), and Pd_2_(dba)_3_ (0.10 g, 0.1 mmol) and p(o-tolyl)_3_ (0.08 g, 0.2 mmol) were added into a two-neck flask containing toluene (20 mL) under argon atmosphere. The mixture was stirred for 24 hours at 140 ^o^C. After cooling to room temperature, the organic solvent of reaction mixtures was removed by reduced pressure and the residue was purified by column chromatography on silica gel using PE:DCM (2:1, *v*/*v*) as the eluent to afford **6** as yellowish powder (1.48 g), yield: 89%. ^1^H NMR (600 MHz, CDCl_3_) δ 8.35 (s, 2H), 8.15 (d, *J* = 1.0 Hz, 2H), 7.14 (s, 2H), 2.67 (d, *J* = 6.9 Hz, 4H), 1.68 (dd, *J* = 16.9, 11.1 Hz, 2H), 1.40 – 1.30 (m, 16H), 0.94 – 0.89 (m, 12H). ^13^C NMR (151 MHz, CDCl_3_) δ 152.99, 144.02, 142.57, 135.08, 132.91, 131.15, 123.77, 109.15, 40.39, 34.61, 32.51, 28.91, 25.67, 23.11, 14.18, 10.89. MALDI-TOF (m/z): [M]^+^ calcd. For C_32_H_40_N_2_O_2_S_2_, 548.8040; found, 549.2602.

**4,8-Bis(5-bromo-4-(2-ethylhexyl)thiophen-2-yl)benzo[1,2-d:4,5-d']bis(oxazole) (M1):** The **6** (1.00 g, 1.8 mmol) were added into a flask containing chloroform (60 mL) in dark. Then the NBS (0.63 g, 3.6 mmol) was added into the mixture in three portions and the reaction was stirred at room temperature for another 0.5 hours. After adding [deionized water](javascript:;) to the flask, the reaction mixture was extracted by DCM. The collected organic layer was removed by reduced pressure and the residue was purified by column chromatography on silica gel using PE:DCM (1:1, *v*/*v*) as eluent to afford **M1** as yellow solid (1.20 g), yield: 94%. ^1^H NMR (600 MHz, CDCl_3_) δ 8.36 (s, 2H), 8.00 (s, 2H), 2.64 (d, *J* = 7.3 Hz, 4H), 1.77 (dd, *J* = 12.3, 6.2 Hz, 2H), 1.44 – 1.30 (m, 16H), 0.94 (dt, *J* = 14.1, 7.3 Hz,12H). ^13^C NMR (151 MHz, CDCl_3_) δ 153.00, 143.70, 141.85, 134.99, 132.83, 130.63, 113.66, 108.54, 40.04, 33.84, 32.50, 28.79, 25.74, 23.10, 14.16, 10.89. Mass (m/z): calcd. For C_32_H_38_Br_2_N_2_O_2_S_2_, 706.5960; found, 707.0787.


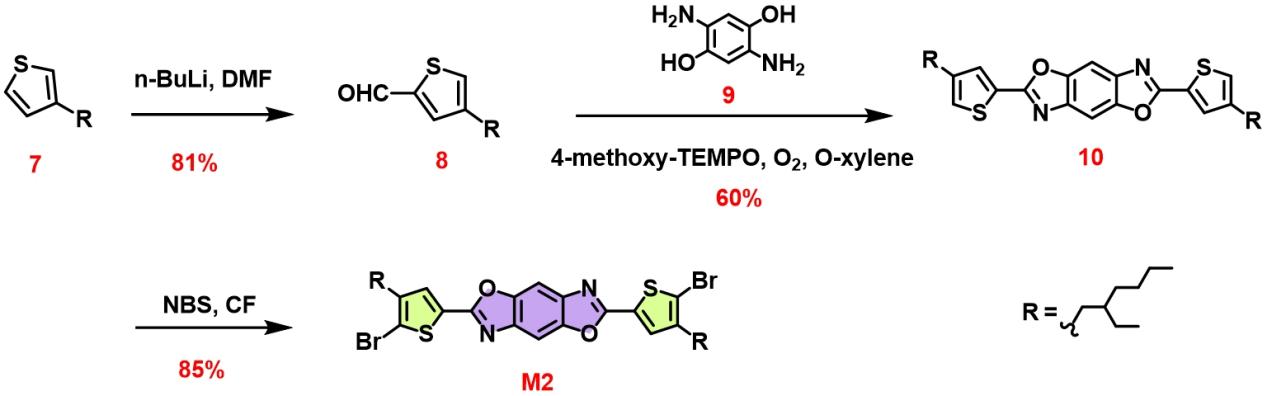


**Scheme S2**. Syntheses of monomer **M2**.

1. **(2-ethylhexyl)thiophene-2-carbaldehyde (8):** To a dry argon filled flask, 7.36 g 3-(2-ethylhexyl) thiophene (37.5 mmol) and 80 mL dry THF were added. The mixture was cooled to -78 °C. While stirring, the *n*-BuLi was added dropwise (17 mL, 2.4 M solution in hexanes, 41.2 mmol) and the reaction was stirred at -78 °C for 2 hours. The 3.8 mL dry N,N-dimethylformamide (48.7 mmol) at -78 °C was added to the reaction mixture, the reaction was allowed to warm to room temperature and stirred overnight. The reaction was quenched with 20 mL water, extracted with petroleum ether, dried over anhydrous Na_2_SO_4_ and concentrated. The residue was purified by chromatography using petroleum ether to give a yellowish oil (7.19 g), yield: 81%. The product was confirmed by ^1^H NMR spectroscopic analysis. ^1^H NMR (600 MHz, Chloroform-d) δ 9.88 (d, J = 1.2 Hz, 1H), 7.59 (s, 1H), 7.35 (s, 1H), 2.59 (d, J = 6.9 Hz, 2H). ^13^C NMR (151 MHz, CDCl_3_) δ 182.97, 143.53, 143.47, 137.66, 131.21, 77.29, 77.08, 76.87, 40.38, 34.15, 32.37, 28.84, 25.51, 22.98, 14.10, 10.80.

**2,6-bis(4-(2-ethylhexyl)thiophen-2-yl)benzo[1,2-d:4,5-d']bis(oxazole) (10):** 2,5-Diaminohydroquinone (DAHQ) (1.40 mg, 10.0 mmol) and 4-(2-ethylhexyl)thiophene-2-carbaldehyde (5.50 g, 2.45 mmol) were reacted in o-xylene solution (15 mL) at 120 ^o^C under argon atmosphere. The mixture was stirred for 5 hours. A water separator is needed to remove water and promote the formation of Schiff base. After cooling to room temperature, 4-methoxy-TEMPO (0.24 g, 1.2 mmol) was added to the mixture, which was stirred at 120 ^o^C for 15 hours under oxygen atmosphere. After removing the solvent under reduced pressure, the obtained crude product was extracted by ethyl ether, washed with distilled water and dried over anhydrous Na_2_SO_4_. The residue was purified by chromatography using petroleum ether-dichloromethane (1:1) to give a colorless crystalline solid (3.61 g), yield: 60 %. The product was confirmed by ^1^H NMR spectroscopic analysis. ^1^H NMR (600 MHz, Chloroform-d) δ 7.85 (s, 1H), 7.79 (s, 1H), 7.19 (s, 1H), 1.67 (s, 3H), 1.32 (s, 9H), 0.93 (s, 6H). ^13^C NMR (151 MHz, CDCl_3_) δ 160.36, 148.23, 143.66, 140.11, 128.67, 126.58, 100.48, 77.23, 77.01, 76.80, 40.43, 34.40, 32.49, 28.92, 25.60, 23.01, 14.14, 10.87. MALDI-TOF (m/z): [M]^+^ calcd. For C_32_H_40_N_2_O_2_S_2_, 548.8040; found, 549.2605.

**2,6-bis(5-bromo-4-(2-ethylhexyl)thiophen-2-yl)benzo[1,2-d:4,5-d']bis(oxazole) (M2):** In a Schlenk flask equipped with a magnetic stirrer, 2,6-bis(4-(2-ethylhexyl)thiophen-2-yl)benzo[1,2-d:4,5-d']bis(oxazole) (0.54 g, 1.0 mmol) and N-bromosuccinimmide (0.44 mg, 2.5 mmol) were dissolved in a mixture of glacial acetic acid (25 mL) and anhydrous N,N-Dimethylformamide (100 mL). The resulting reaction mixture was shielded from light and stirred for 48 hours at room temperature. The solvent was then removed under reduced pressure and the residue was purified by column chromatography using petroleum ether-dichloromethane (3:1) to give a yellow solid (0.61 g), yield: 85%. The product was confirmed by ^1^H and ^13^C NMR spectroscopic analysis. 1H NMR (600 MHz, Chloroform-d) δ 7.81 (s, 2H), 7.61 (s, 2H), 2.58 (s, 4H), 1.67 (s, 4H), 1.37 (s, 10H), 0.94 (s, 18H). ^13^C NMR (151 MHz, CDCl_3_) δ 159.31, 148.21, 143.15, 140.21, 131.26, 128.43, 116.02, 100.61, 77.23, 77.02, 76.81, 39.99, 33.81, 32.51, 28.82, 25.66, 23.02, 14.12, 10.82. Mass (m/z): calcd. For C_32_H_38_Br_2_N_2_O_2_S_2_, 706.5960; found, 707.0791.


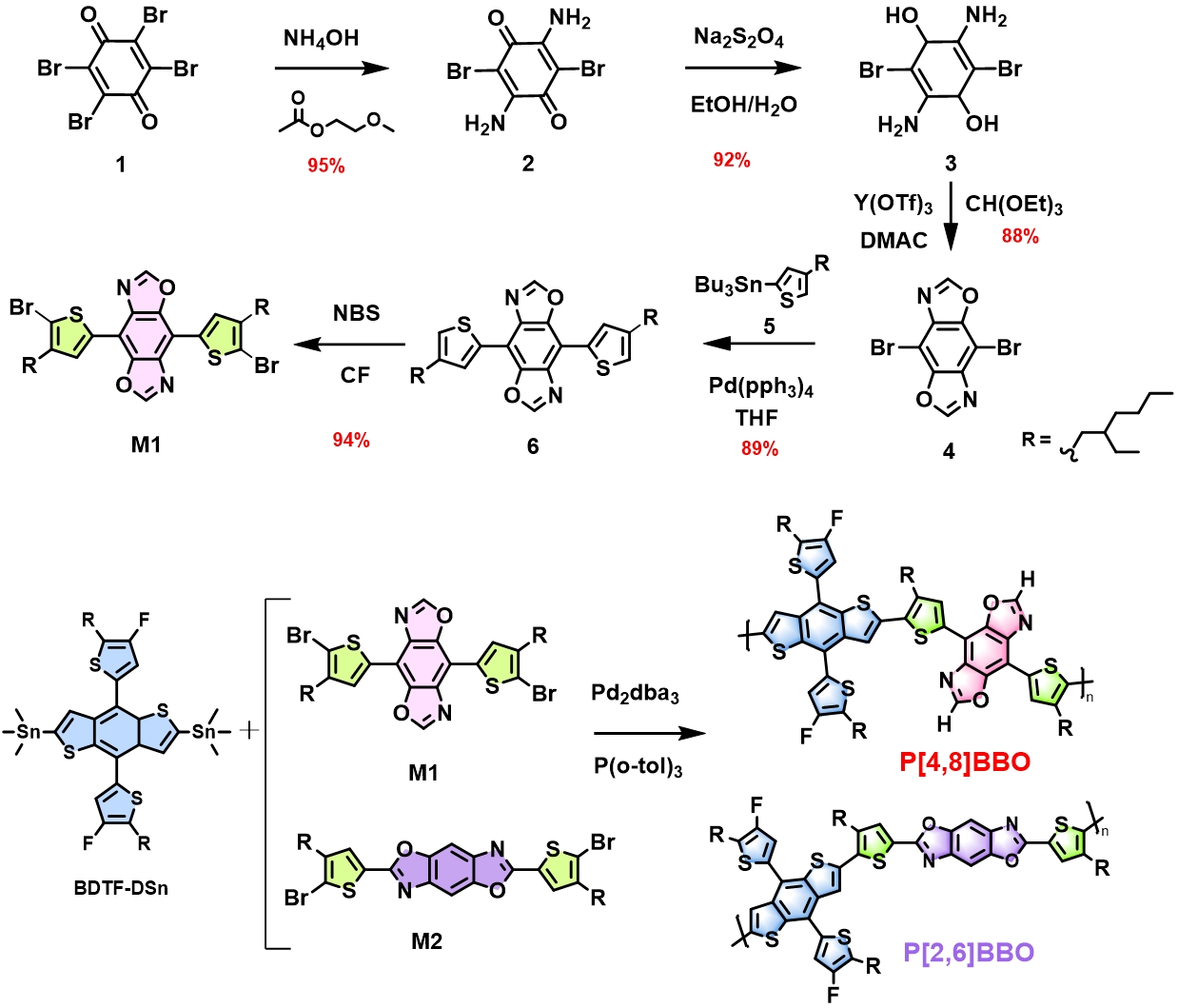


**Scheme S3.** The synthetic routes of polymer P[4,8]BBO and P[2,6]BBO.

**Polymerization of P[4,8]BBO**: **BDTF-DSn** (188.10 mg, 0.20 mmol), **M1** (141.30 mg, 0.20 mmol), Pd_2_(dba)_3_ (10.02 mg) and P(*o*-tol)_3_ (15.03 mg) were combined in a 48 mL sealed tube. Dry chlorobenzene (CB) (12 mL) was added under argon atmosphere. The mixture was reacted at 80 ^o^C for 24 hours and then 100 ^o^C for 48 hours. After cooled down to 50 ^o^C, the reactant mixture was poured into MeOH (500 mL). The precipitate was filtered and Soxhlet extracted with methanol, hexane, dichloromethane, and chloroform. The chloroform ingredient was concentrated, precipitated into 500 mL methanol, and dried under vacuum to afford the purple fiber P[4,8]BBO (197.5 mg, yield 85%). ^1^H NMR (600 MHz, C_2_D_2_Cl_4_) δ 8.40 (s, 2H), 8.25 (s, 2H), 7.82 (s, 2H), 7.30 (s, 2H), 2.96 (m, 4H), 2.89 (m, 4H), 1.88 (m, 4H), 1.32 (m, 16H), 1.09 – 0.88 (m, 40H). Molecular weight: *M_n_* = 58.9 kDa, *M_w_* = 143.0 kDa, PDI = 1.84.

**Polymerization of P[2,6]BBO**: **BDTF-DSn** (148.42 mg, 0.16 mmol), **M2** (111.5 mg, 0.16 mmol), Pd_2_(dba)_3_ (10.01 mg) and P(*o*-tol)_3_ (15.02 mg) were combined in a 48 mL sealed tube. Dry chlorobenzene (CB) (12 mL) was added under argon atmosphere. The mixture was reacted at 80 ^o^C for 24 hours and then 100 ^o^C for 48 hours. After cooled down to 50 ^o^C, the reactant mixture was poured into MeOH (500 mL). The precipitate was filtered and Soxhlet extracted with methanol, hexane, dichloromethane, and chloroform. The chloroform ingredient was concentrated, precipitated into 500 mL methanol, and dried under vacuum to afford the purple fiber P[2,6]BBO (168.64 mg, yield 80%). ^1^H NMR (600 MHz, C_2_D_2_Cl_4_) δ 7.95 – 7.52 (m, 4H), 7.35 (m, 4H), 2.88 (m, 8H), 1.80 (m, 4H), 1.30 (m, 32H), 0.94 (m, 24H). Molecular weight: *M_n_* = 37.3 kDa, *M_w_* = 65.9 kDa, PDI = 1.77.

# 3. Gel Permeation Chromatography Measurements

The molecular weights of polymers were obtained on an Acquity Advanced Polymer Chromatography (Waters) with a high-temperature chromatograph in 1,2,4-trichlorobenzene at 150 ^o^C and using a calibration curve of polystyrene standards.


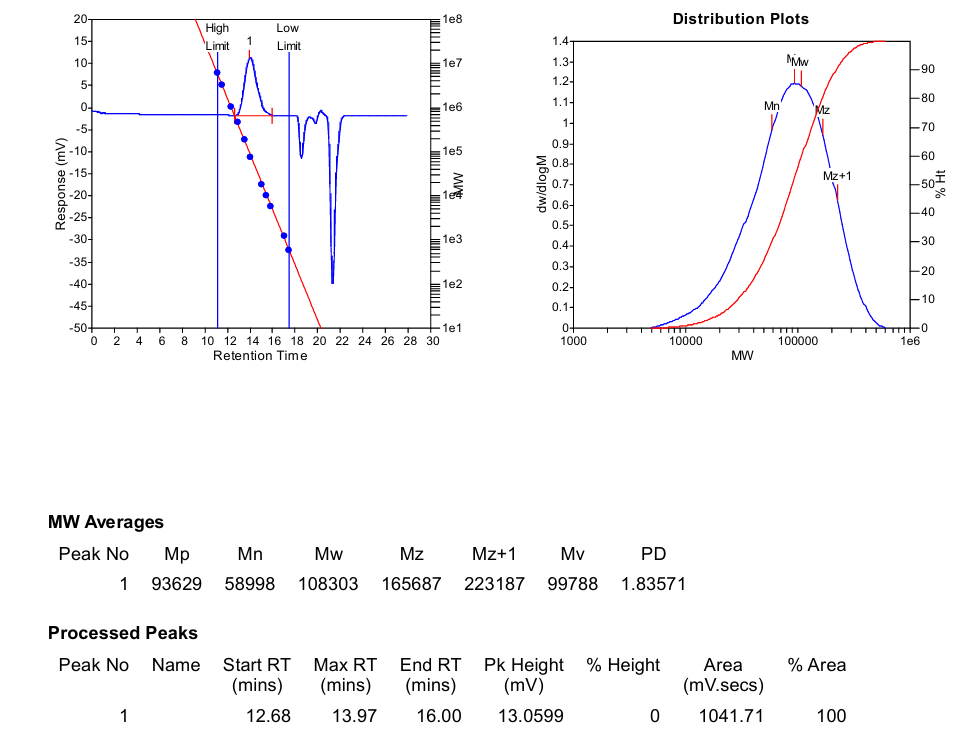


**Figure S3.** High-temperature GPC measurement of P[4,8]BBO.


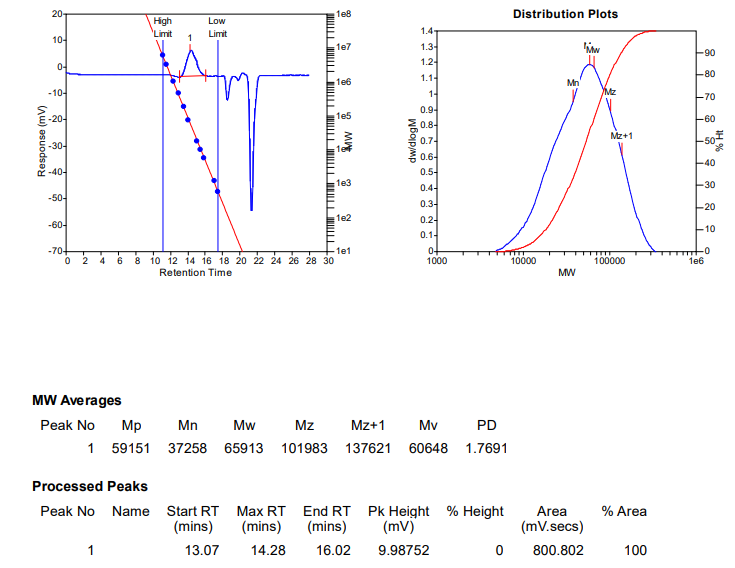


**Figure S4.** High-temperature GPC measurement of P[2,6]BBO.

**Table S3**. High-temperature GPC data of P[4,8]BBO and P[2,6]BBO.

| Material | Mn | Mw | PDI |
| --- | --- | --- | --- |
| P[4,8]BBO | 58998 | 108303 | 1.84 |
| P[2,6]BBO | 37258 | 65913 | 1.77 |

# 4. UV−Vis Absorption Spectra

The absorption spectra were measured using a SHIMADZU UV-3600 spectrophotometer in the range of 300–1000 nm, with corrections for quartz absorption. For solution absorption measurements, samples were dissolved in CF solvent at a concentration of 10^-5^ mol/L. For film absorption measurements, ~100 nm thick films were prepared by spin-coating the CF solution onto quartz plates at an optimized speed.

**Table S4**. Ratio of the 0-0 peak intensity to the 0-1 peak intensity (*I_(0-0)_/I_(0-1)_*) from temperature-dependent absorption spectra for P[2,6]BBO and P[4,8]BBO.

| Material | 25℃  % | 30℃  % | 40℃  % | 50℃  % | 60℃  % | 70℃  % | 80℃  % | 90℃  % | 100℃  % |
| --- | --- | --- | --- | --- | --- | --- | --- | --- | --- |
| P[2,6]BBO | 143.25 | 143.02 | 140.15 | 136.45 | 132.02 | 127.61 | 123.37 | 119.72 | 114.03 |
| P[4,8]BBO | 106.70 | 106.21 | 104.44 | 102.08 | 98.64 | 94.40 | 89.35 | 82.88 | 71.90 |

# 5. Photoelectron Spectroscopy in Air (PESA) Measurements

The photoelectron spectroscopy in air (PESA) measurements were recorded using a RIKEN KEIKI spectrometer (Model AC-3) with a power setting of 30 nW and a power number of 0.5. Samples for PESA measurements were prepared on glass substrate.

# 6. Photoluminescent (PL) spectra

Photoluminescence (PL) data were collected using the HORiBA FLUOROMAX-4 fluorimeter. The wavelength was set to 530 nm for P[4,8]BBO and P[2,6]BBO excitation, and 840 nm for eC9-2Cl excitation.

# 7. OSCs Fabrication and Characterization

## 7.1. Device Fabrication.

The conventional structure of ITO/PEDOT:PSS/2PACZ/Polymer_Donor:eC9-2Cl/PNDIT-F_3_N/Ag was used to fabricate the OSCs. The indium tin oxide (ITO) substrates were cleaned sequentially by sonication with detergent, deionized water (DIW), and isopropanol. After being dried in an oven at 60 ℃ overnight, the substrates were treated with an oxygen plasma for 5 min and then coated with PEDOT:PSS (CLEVIOS PVP Al 4083) and 2PACZ at 3000 rpm for 30 s as hole transport layer (HTL). The blend solutions, with a donor-to-acceptor ratio of 1:1.8 (w/w) were prepared by dissolving them in chloroform. The total concentration was fixed to 18 mg mL^−1^ when optimizing the donor:acceptor ratios. Subsequently, these solutions were spin-coated onto HTL layer to forming active layer. A 5 nm PNDIT-F_3_N (1 mg mL^−1^ in MeOH) was then spin-coated onto the active layers as a cathode interface. Finally, 100 nm silver was thermally deposited on top of the interface through a shadow mask in a vacuum chamber at a pressure of 1×10^−7^ mbar. The effective area of the device was confined to 0.04 cm^2^ by a non-refractive mask to improve the accuracy of measurements.

## 7.2 Photovoltaic Performance of Benzobisazole based Donors.

**Figure S5.** The chemical structure of benzobisazole-based polymeric and small molecular donors.

**Table S5**. The photovoltaic performances of the benzobisazole (BBO)-based donors.

| Active layer materials | *V*_OC_  [V] | *J*_SC_  [mA cm^−2^] | FF  [%] | PCE  [%] | References |
| --- | --- | --- | --- | --- | --- |
| P1:PC_71_BM | 0.74 | 4.22 | 31.48 | 0.98 | *Macromolecules***2011***, 44,* 9611–9617 |
| P2:PC_71_BM | 0.73 | 5.13 | 30.6 | 1.14 |  |
| P3:PC_71_BM | 0.72 | 13.96 | 45.0 | 3.51 | *J. Polym. Sci. Polym. Chem.,* **2015***, 53,* 1533-1540 |
| P4:PC_71_BM | 0.71 | 5.13 | 46.0 | 1.67 |  |
| P5:PC_71_BM | 0.76 | 4.52 | 53.0 | 1.78 | *J. Polym. Sci. Polym. Chem.,* **2016***, 54* 316-324 |
| P6:PC_71_BM | 0.72 | 7.81 | 49.0 | 2.74 |  |
| PM6:PBBO: BTP-eC9  Ternary OSCs | 0.85 | 27.8 | 78.1 | 18.5 | ***Nano Energy,* 2024, *126*, 109648** |
| P[4,8]BBO:eC9-2Cl | **0.87** | **28.1** | **77.5** | **19.0** | **This work** |

**Table S6**. The photovoltaic performance of the representative all-PSCs and their related certified efficiencies.

| Active layer materials | *V*_OC_  [V] | *J*_SC_  [mA cm^−2^] | FF  [%] | PCE  [%] | Certified PCE  [%] | References |
| --- | --- | --- | --- | --- | --- | --- |
| PM6:PYT:PY2F-T | 0.90 | 25.2 | 24.6 | 17.2 | 17.0 | *Joule.* 2021, *5*, 1548-1565. |
| PM6:PY-IT:PYCl-T | 0.92 | 24.6 | 73.3 | 16.6 | 16.3 | *Sci. China Chem.* 2022*, 65*, 954–963 |
| PM6:PY-DT | 0.95 | 23.7 | 74.4 | 16.8 | 16.3 | ***Adv. Mater.* 2022, *34*, 2110155.** |
| PBQ6:PYF-T-o | 0.89 | 25.1 | 76.6 | 17.1 | 16.6 | ***Energy Environ. Sci.*** 2022, ***15***, 4157-4166 |
| PM6:PY-82 | 0.95 | 23.8 | 75.8 | 17.2 | 16.7 | ***Energy Environ. Sci.*** 2022, ***15***, 3854-3861 |
| PM6：PY-1S1Se:PY-2Cl | 0.91 | 25.7 | 77.2 | 18.2 | 17.7 | *Joule.* 2023, *7*, 221-237 |
| PM6:PY-IT | 0.95 | 26.4 | 76.4 | 19.1 | 18.6 | *Nat. Commun.* 2023, *14*, 4148 |
| PM6:PY-DT:PYF-T-*o* | 0.95 | 24.6 | 78.1 | 18.2 | 17.7 | *Matter.* 2023, *6*, 1542-1554 |
| PQB-2:PY-IT | 0.94 | 24.2 | 79.5 | 18.1 | 17.6 | ***Energy Environ. Sci.***, 2023, ***16***, 1581-1589 |
| PM6:PY-82:PY-DT | 0.95 | 24.3 | 78.3 | 18.0 | 17.5 | *Adv. Mater,* 2023, *35*, 2208165. |
| PM6:PY-DT-X(2-EN) | 0.95 | 25.9 | 79.1 | 19.5 | 19.1 | *Adv. Mater,* 2024, *36*, 2406922. |
| PM6:PBQx-TF:PY-IT | 0.95 | 25.5 | 79.9 | 19.40 | 19.07 | *Adv. Mater,* 2024, *37*, 2411989. |
| PM6:PY-IT | 0.94 | 26.1 | 78.6 | 19.2 | 19.1 | ***Angew. Chem. Int. Ed.* 2025*,* e202425267** |
| P[4,8]BBO:PM6:PY-IT | **0.91** | **26.6** | **79.7** | **19.4** | **19.1** | **This work** |

#

# 8. Fabrication and Characterization of SCLC Devices

The charge carrier mobilities of pristine and blend films were estimated using the space-charge-limited current (SCLC) method. Hole-only and electron-only devices were fabricated with the architectures ITO/PEDOT:PSS/Active Layer/MoO₃/Ag and ITO/ZnO/Active Layer/PFNDIT-F3N/Ag, respectively. The current–voltage characteristics of these devices were measured in the dark using a Keithley 236 source meter. The hole and electron mobilities were extracted by fitting the dark current curves to the single-carrier SCLC model, described by the following equation:

$$J=\frac{9}{8}\varepsilon_{0}\varepsilon_{\gamma}\mu\frac{V^{2}}{d^{3}}$$

where *J* is the current density, *μ* is the zero-field mobility, *ε*_0_ is the permittivity of free space, *ε*_r_ is the relative permittivity of the material, *d* is the thickness of the active layers, and *V* is the effective voltage. The effective voltage was obtained by subtracting the built-in voltage (*V*_bi_) and the voltage drop (*V*_s_) from the series resistance of the whole device except for the active layers from the applied voltage (*V*_appl_), *V* = *V*_appl_ − *V*_bi_ − *V*_s_ (*V*_bi_ = 0 and *V*_s_ =10×*I*, where the value 10 is the resistance of MoO_3_ and *I* is the current of the devices in this work). The hole and electron mobilities can be calculated from the slope of the *J*^1/2^*-V* curves.


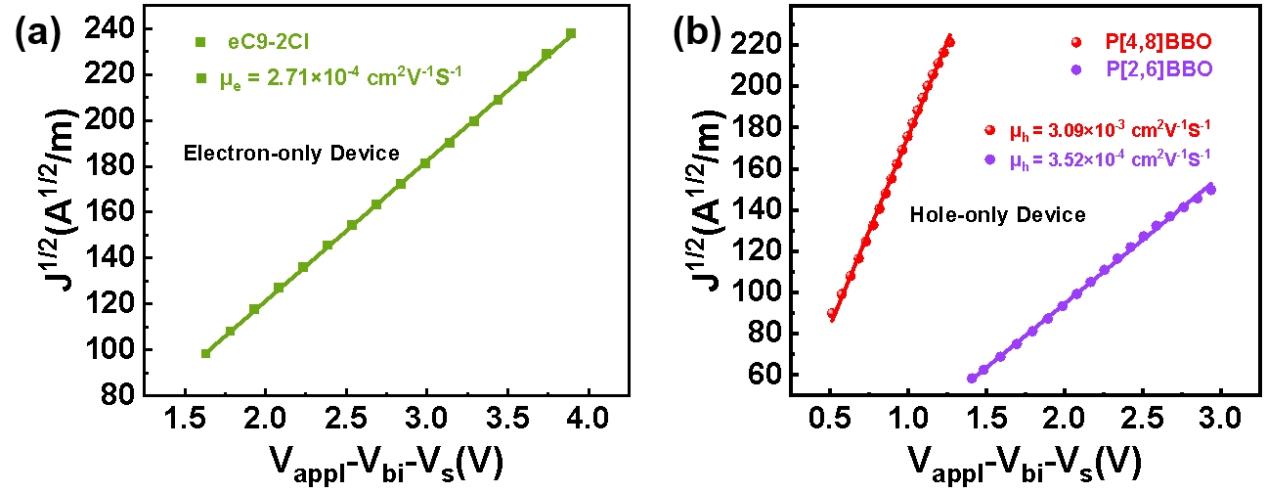


**Figure S6.** SCLC plots of the electron-only and hole-only devices: (a) pristine eC9-2Cl film; (b) pristine P[2,6]BBO and P[4,8]BBO films.

# 9. D Grazing Incidence Wide Angle X-ray Scattering (GIWAXS)

2D-GIWAXS measurement was performed on an XEUSS 3.0 UHR WAXS system (XENOCS, France). A Eiger2 R 1M 2-dimensional detector with 0.075 mm×0.075 mm active pixels was utilized in integration mode. The detector was positioned about 100/2000 mm downstream from the sample location. The precise sample-to-detector distance was determined with a silver behenate standard. The Cu incident X-ray (8 KeV) with a 0.9 mm×0.9 mm/0.5 mm×0.5 mm spot provided large enough q space. 1D GIWAXS patterns was corrected to represent real q_r_ and q_xy_ axes with the consideration of missing wedge. The critical incident angle was determined by the maximized scattering intensity from sample scattering with negligible contribution from underneath layer scattering. The shallow incident angle scattering was collected at 0.2°, which renders the incident X-ray as an evanescent wave along the top surface of thin films. The samples for GIWAXS test were prepared by casting solution onto silicon wafer substrates (ca. 15 mm×15 mm), and the active layers were prepared using same concentration and procedures as those for *J–V* measurements.

**Table S7.** GIWAXS parameters of pristine P[4,8]BBO, P[2,6]BBO, and eC9-2Cl films in IP direction.

| Sample | IP (100) | | | |
| --- | --- | --- | --- | --- |
|  | *q*  [Å^-1^] | *d*-spacing  [Å] | FWHW  [Å^-1^] | CCL  [Å] |
| P[4,8]BBO | 0.315 | 19.97 | 0.103 | 55.1 |
| P[2,6]BBO | 0.281 | 22.38 | 0.110 | 51.5 |
| eC9-2Cl | 0.396 | 15.86 | 0.089 | 63.1 |

**Table S8.** GIWAXS parameters of P[4,8]BBO:eC9-2Cl and P[2,6]BBO:eC9-2Cl films in IP direction.

| Sample | IP (100) | | | |
| --- | --- | --- | --- | --- |
|  | *q*  [Å^-1^] | *d*-spacing  [Å] | FWHW  [Å^-1^] | CCL  [Å] |
| P[4,8]BBO:eC9-2Cl | 0.344 | 18.28 | 0.079 | 71.2 |
| P[2,6]BBO:eC9-2Cl | 0.282 | 22.25 | 0.045 | 125.1 |

**Table S9.** GIWAXS parameters of pristine P[4,8]BBO, P[2,6]BBO, and eC9-2Cl films in OOP direction.

| Sample | OOP (010) | | | |
| --- | --- | --- | --- | --- |
|  | *q*  [Å^-1^] | *d*-spacing  [Å] | FWHW  [Å^-1^] | CCL  [Å] |
| P[4,8]BBO | 1.597 | 3.93 | 0.285 | 19.9 |
| P[2,6]BBO | 1.678 | 3.75 | 0.171 | 33.1 |
| eC9-2Cl | 1.674 | 3.75 | 0.344 | 16.4 |

**Table S10.** GIWAXS parameters of P[4,8]BBO:eC9-2Cl and P[2,6]BBO:eC9-2Cl films in OOP direction.

| Sample | OOP (010) | | | |
| --- | --- | --- | --- | --- |
|  | *q*  [Å^-1^] | *d*-spacing  [Å] | FWHW  [Å^-1^] | CCL  [Å] |
| P[4,8]BBO:eC9-2Cl | 1.673 | 3.76 | 0.315 | 18.0 |
| P[2,6]BBO:eC9-2Cl | 1.769 | 3.55 | 0.155 | 36.4 |

# 10. Atom Force Microscopy (AFM) Measurement

Atomic force microscopy (AFM) of the blended film was conducted using a Digital Instruments DI Multimode Nanoscope III in tapping mode. The samples for the AFM measurements were prepared under the same conditions as the OSC devices, but without the cathode interface layer and Ag electrode.


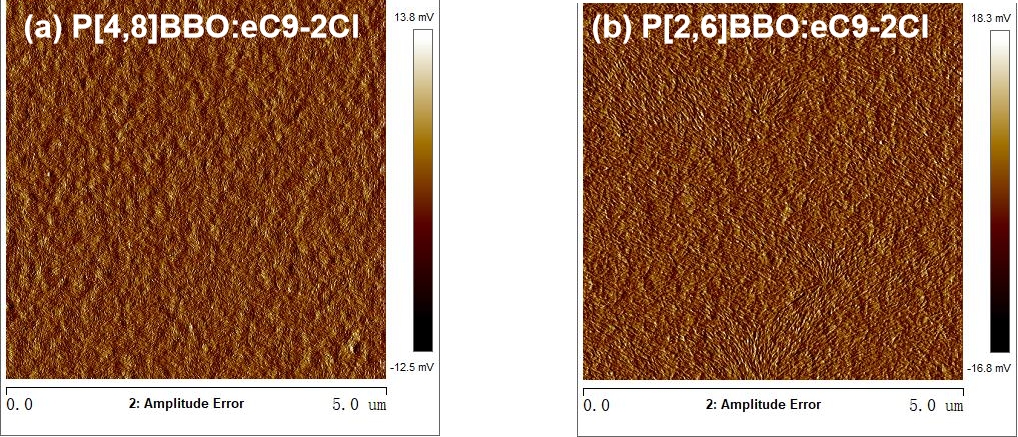


**Figure S7.** AFM phase images：(a) P[4,8]BBO:eC9-2Cl and (b) P[2,6]BBO:eC9-2Cl based BHJ active layers.

# 11.Transmission Electron Microscopy (TEM) Measurement

The transmission electron microscopy (TEM) characterization was carried out using a JEM-2100F instrument. The samples for the TEM measurements were prepared as follows: the active-layer films were spin-cast onto ITO/PEDOT:PSS-coated substrates, and the substrates with the active layers were submerged in deionized water to make the active layers float on the air-water interface. Then, the floated films were picked up on unsupported 200 mesh copper grids for the TEM measurements.

# 12. Ultra-fast Transient Absorption Spectroscopy Measurements

Transient absorption measurement was conducted on a commercial pump-probe femtosecond transient absorption (TA) spectrometer Helios (Ultrafast System, USA). Ultrafast laser pulses (800 nm, < 35 fs pulse duration, 7 W) was generated by 1 kHz Ti:Sapphire regenerative amplifier (Astrella, Coherent, USA). 40% of the fundamental pulses (7 W) was used to pump the commercial collinear optical parametric amplifier (TOPAS Prime, Light-Conversion, Lithuania) for generating tunable wavelength pump pulse to 400 nm or 800 nm. The pump beam is chopped at 500 Hz. 15% of the fundamental pulses was routed onto a mechanical delay stage (within 7 ns) and passed through a sapphire crystal to generate supercontinuum probe light (450-750 nm). The pump light and probe light were focused on the same spot (2 mm diameter) of the thin films placed on a quartz. Data analysis is performed by Surface Xplorer software. The incident power is measured with a calibrated laser power meter (Newport).


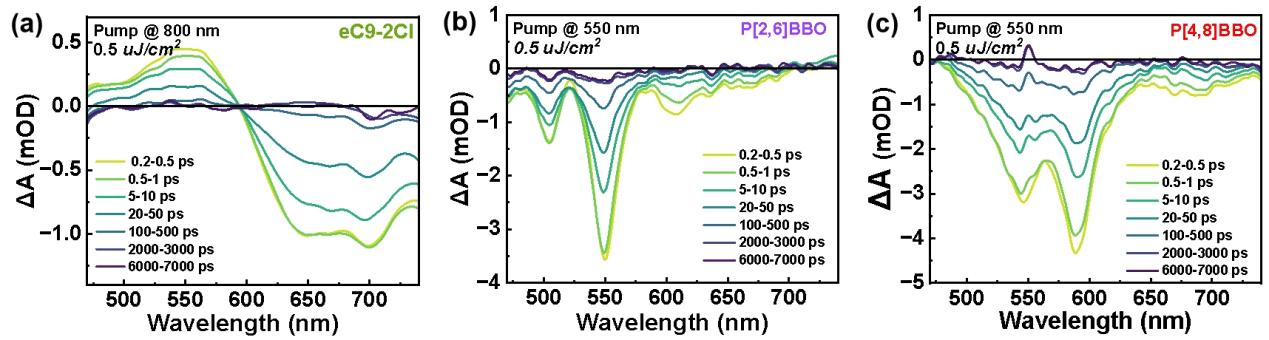


**Figure S8.** The ultra-fast transient absorption spectra of the (a) eC9-2Cl, (b) P[2,6]BBO, (c) P[4,8]BBO based neat films.

# 13. Certified Report of P[4,8]BBO:PM6:PY-IT-based all-PSCs.


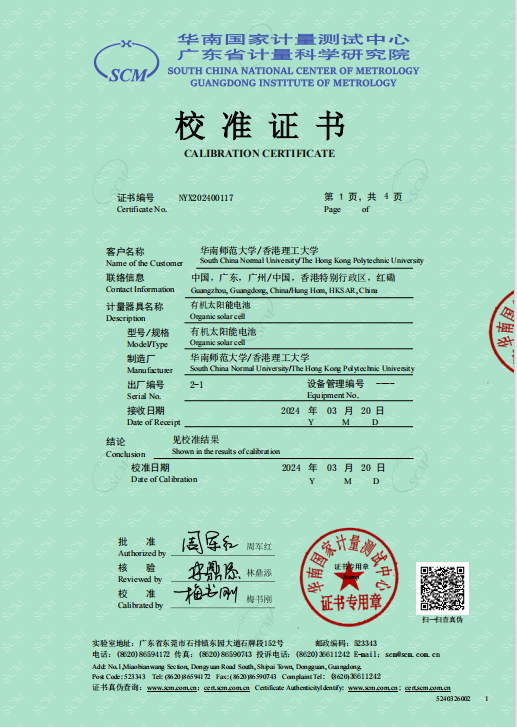


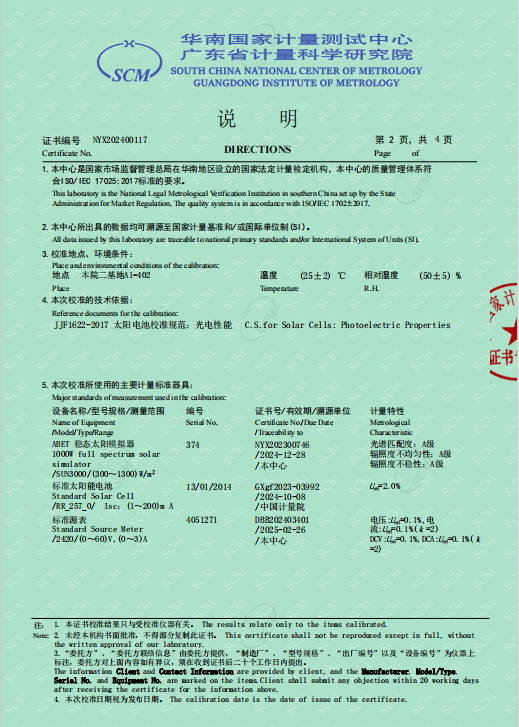


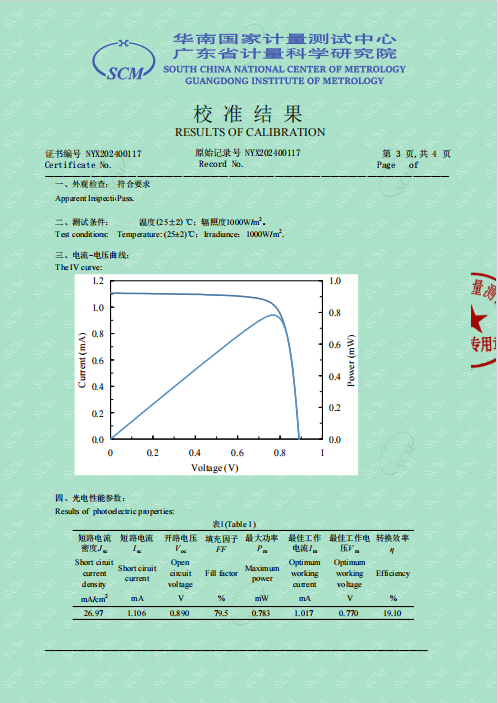


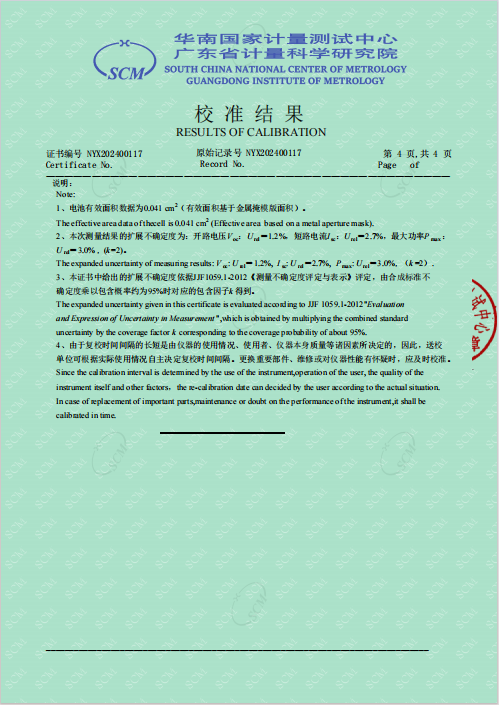


**Figure S9**. The certified efficiency of P[4,8]BBO-based all-polymer solar cells.

# 14. Solution NMR and Mass Spectra

The ^1^H NMR and ^13^C NMR spectra were measured on a Bruker AVANCE NEO (600 MHz) spectrometer with tetramethylsilane (TMS) as the internal reference at room temperature. Mass spectra were measured on Bruker ultrafleXtreme instrument.


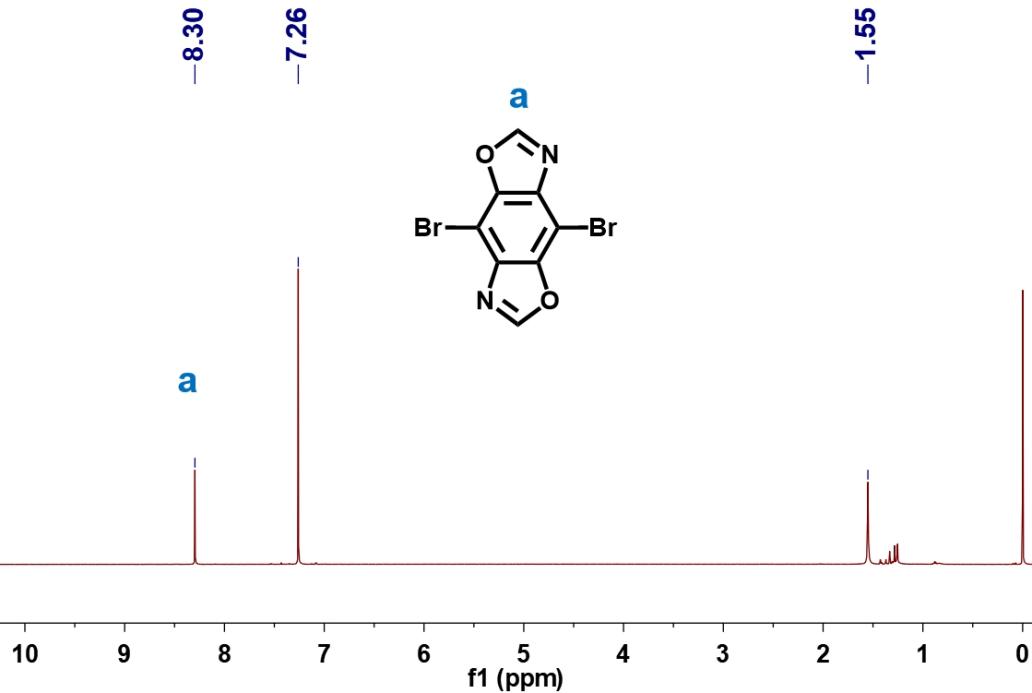


**Figure S10.** The ^1^H NMR spectrum of compound **4** in CDCl_3_ at room temperature.


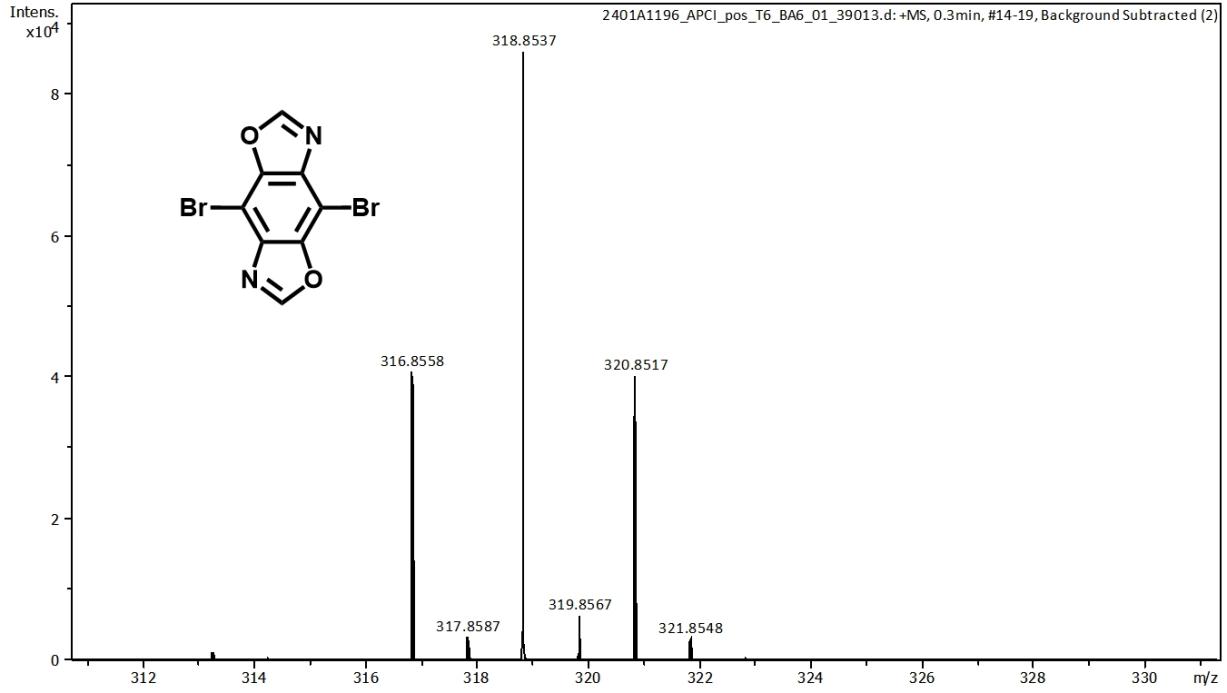


**Figure S11.** The MS spectrum of compound **4**.


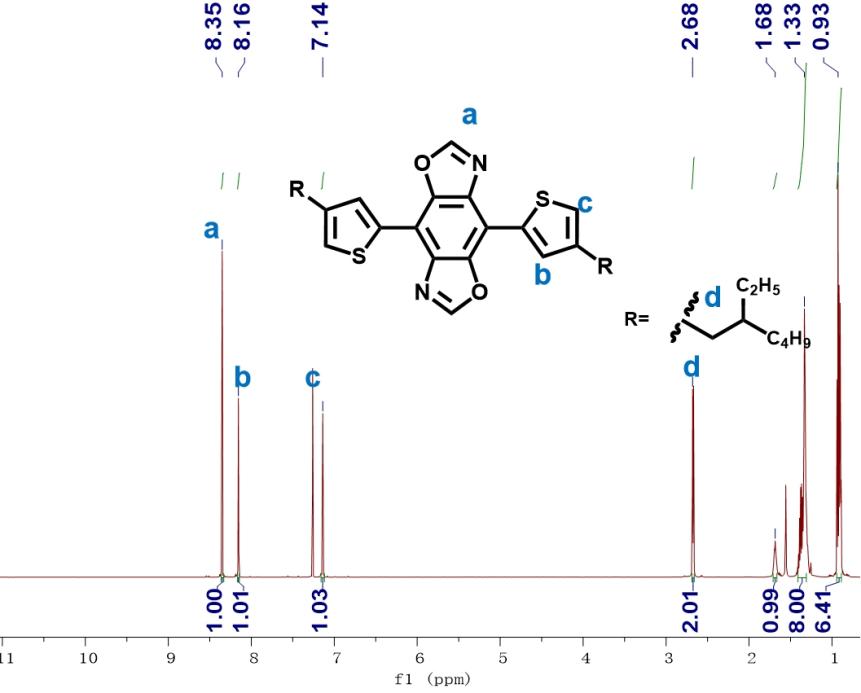


**Figure S12.** The ^1^H NMR spectrum of compound **6** in CDCl_3_ at room temperature.


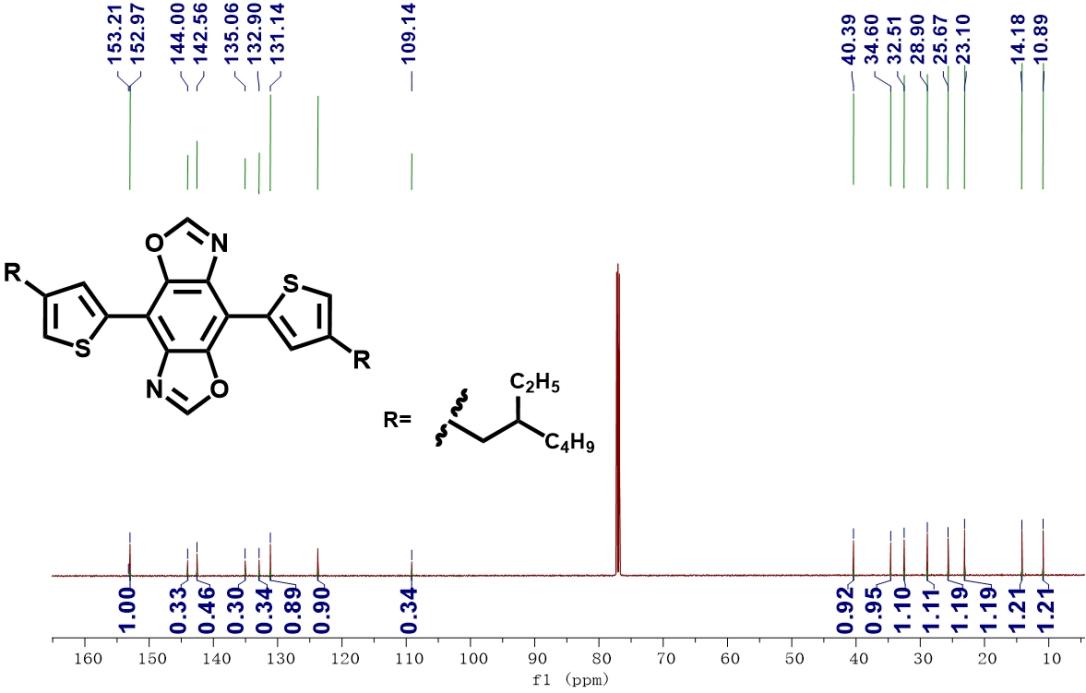


**Figure S13.** The ^13^C NMR spectrum of compound **6** in CDCl_3_ at room temperature.


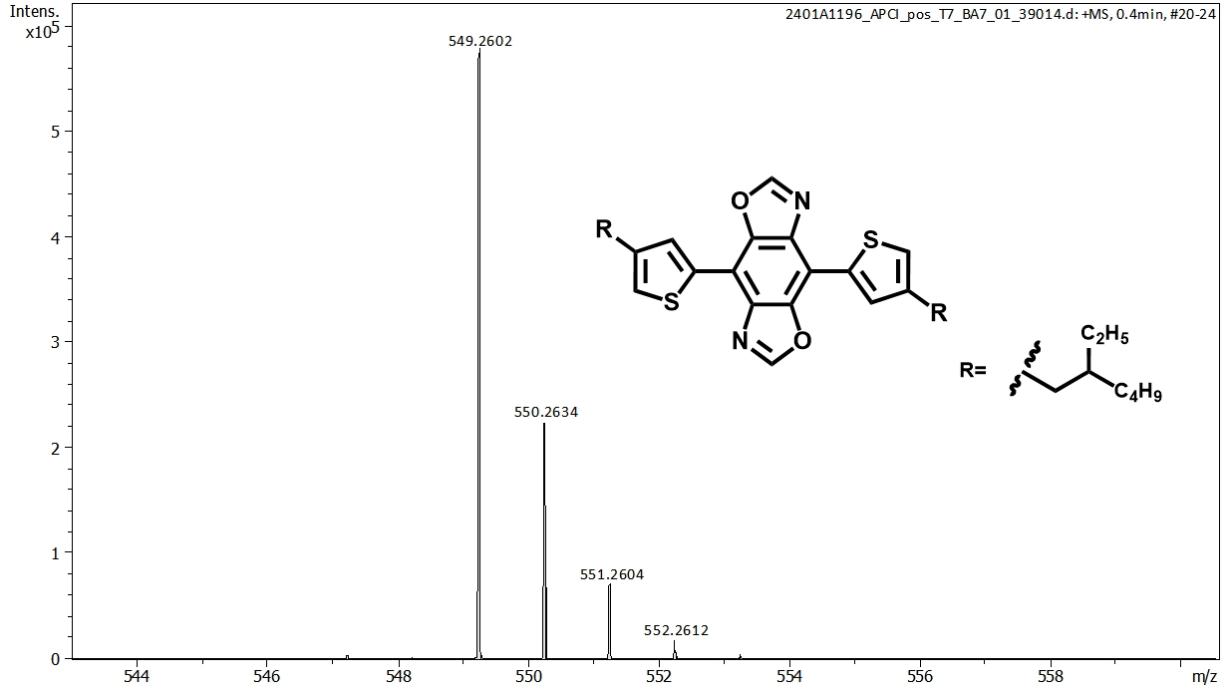


**Figure S14.** The MS spectrum of compound **6**.


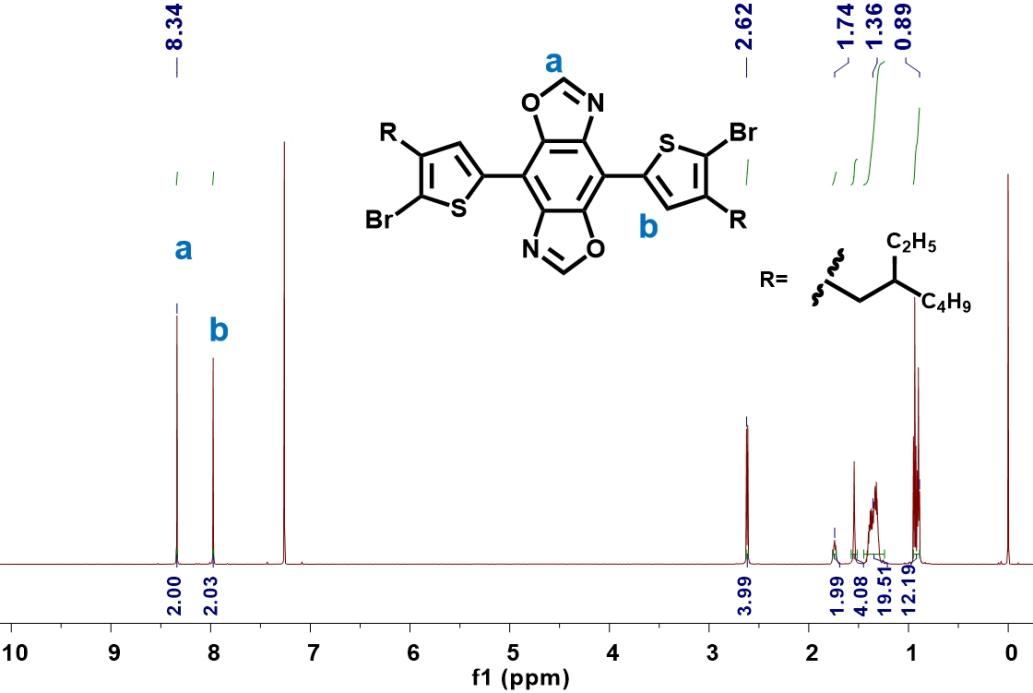


**Figure S15.** The ^1^H NMR spectrum of **M1** in CDCl_3_ at room temperature.


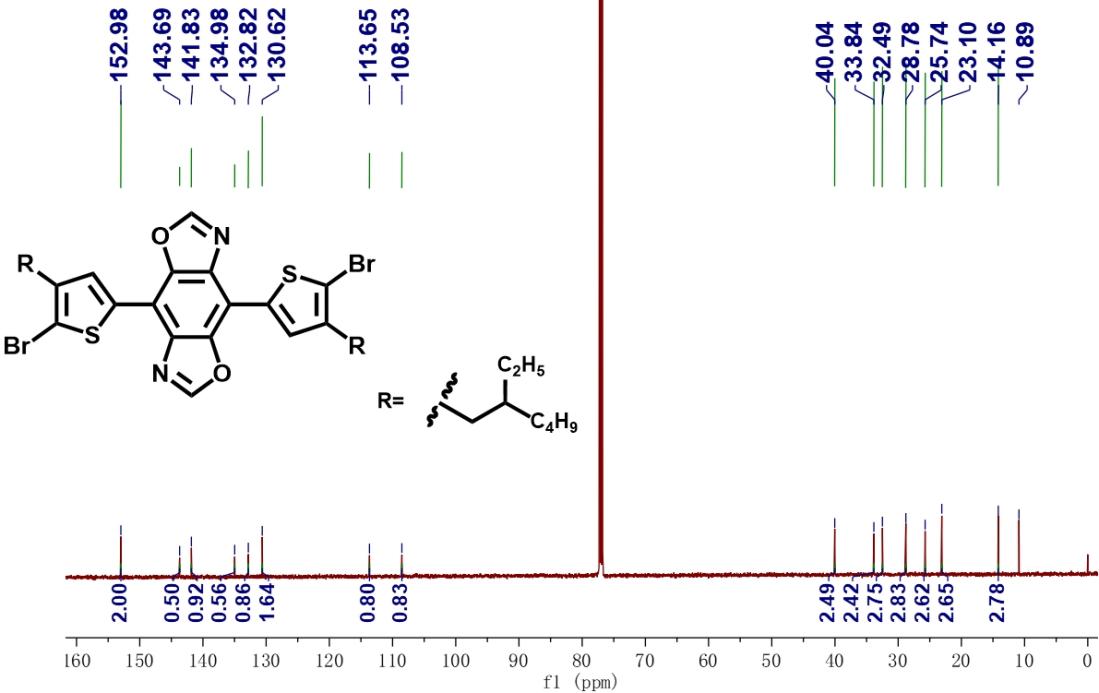


**Figure S16.** The ^13^C NMR spectrum of **M1** in CDCl_3_ at room temperature.


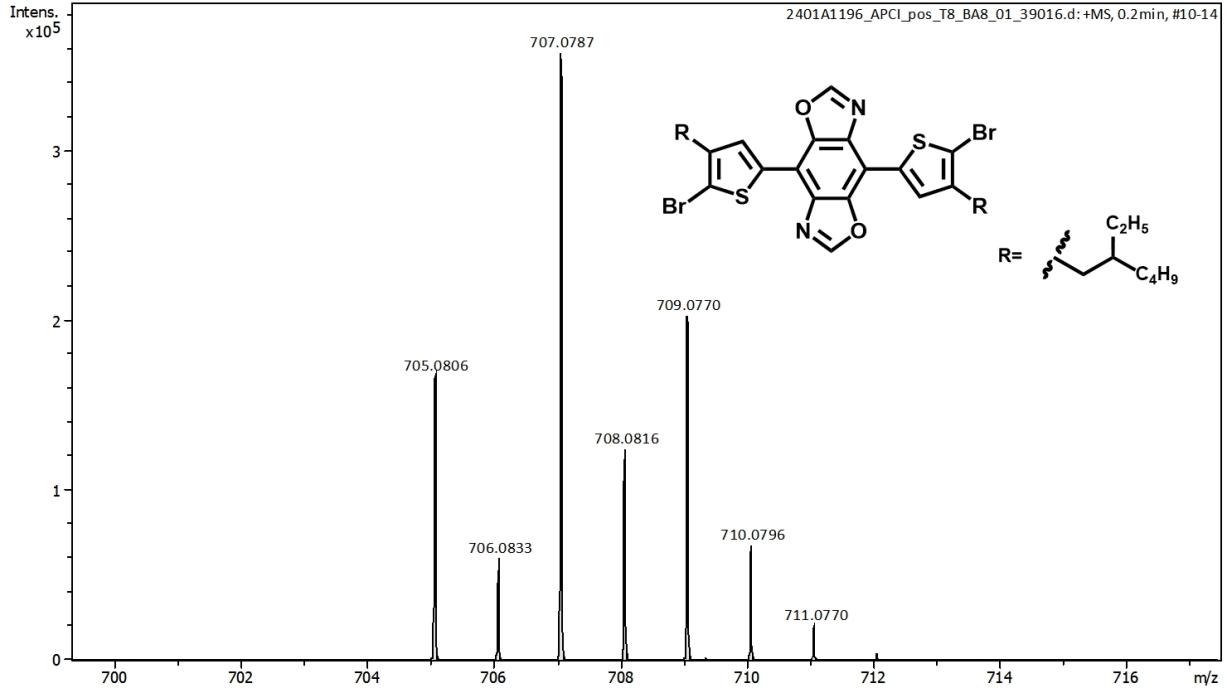


**Figure S17.** The MS spectrum of **M1**.


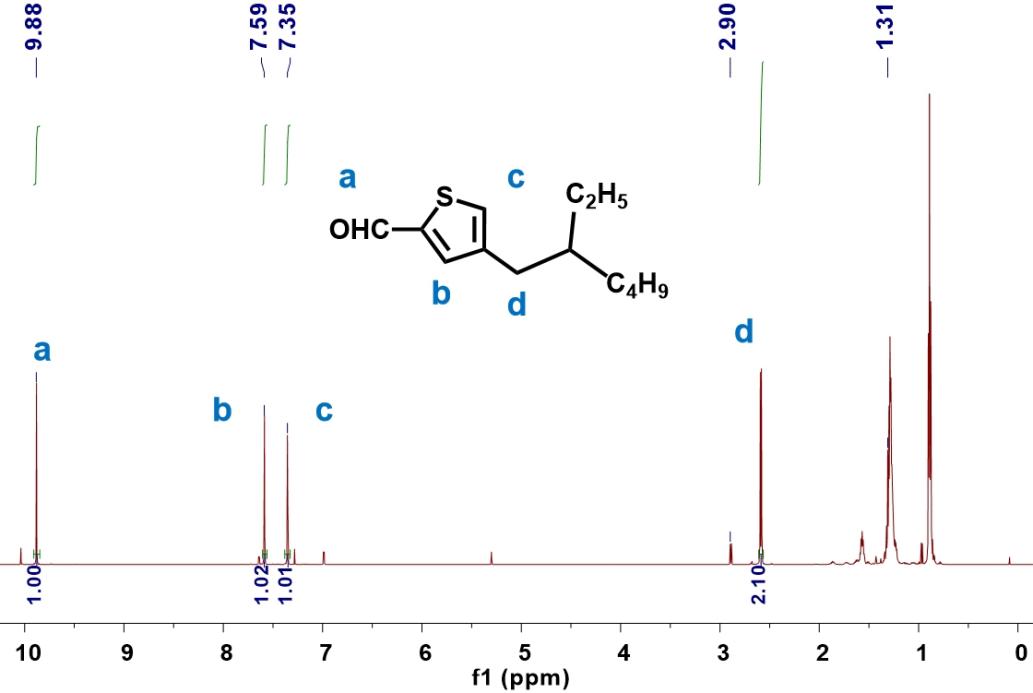


**Figure S18.** The ^1^H NMR spectrum of compound **8** in CDCl_3_ at room temperature.


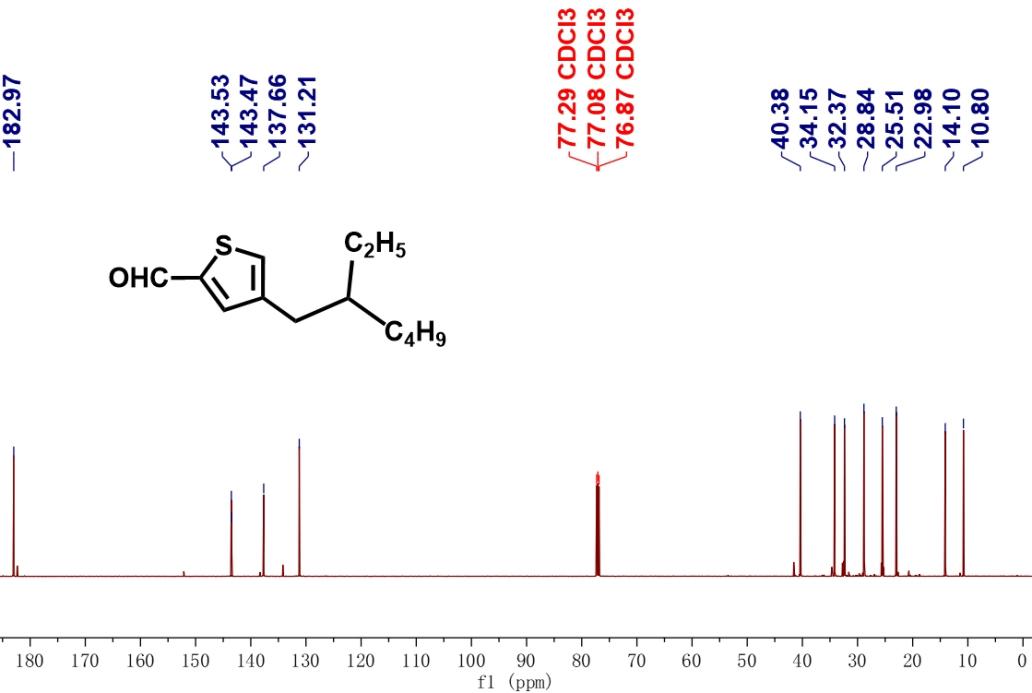


**Figure S19.** The ^13^C NMR spectrum of compound **8** in CDCl_3_ at room temperature.


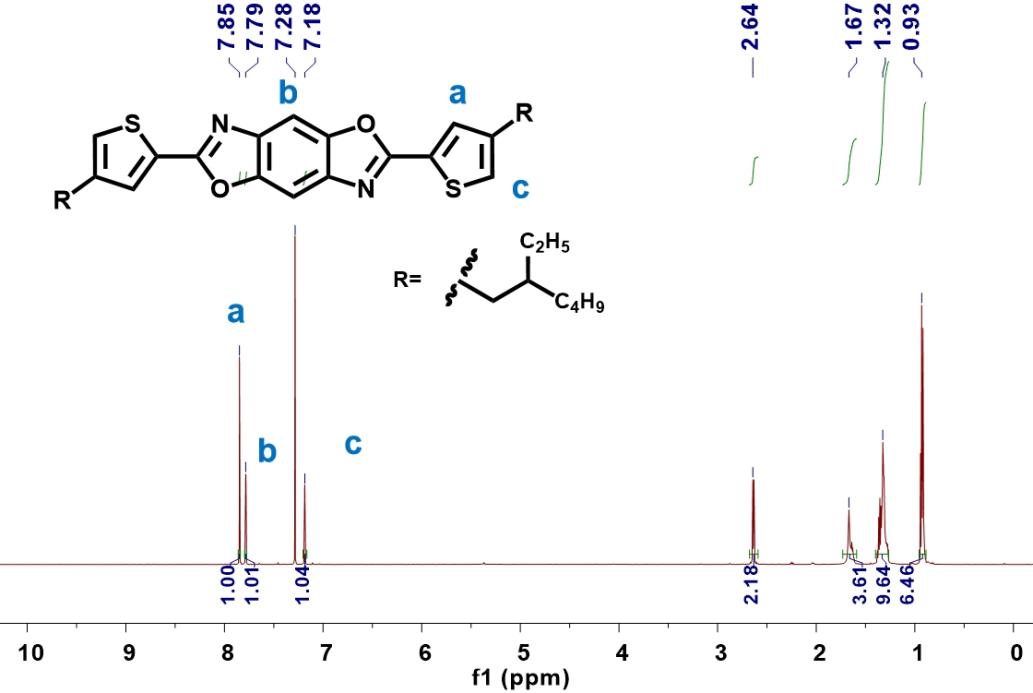


**Figure S20.** The ^1^H NMR spectrum of compound **10** in CDCl_3_ at room temperature.


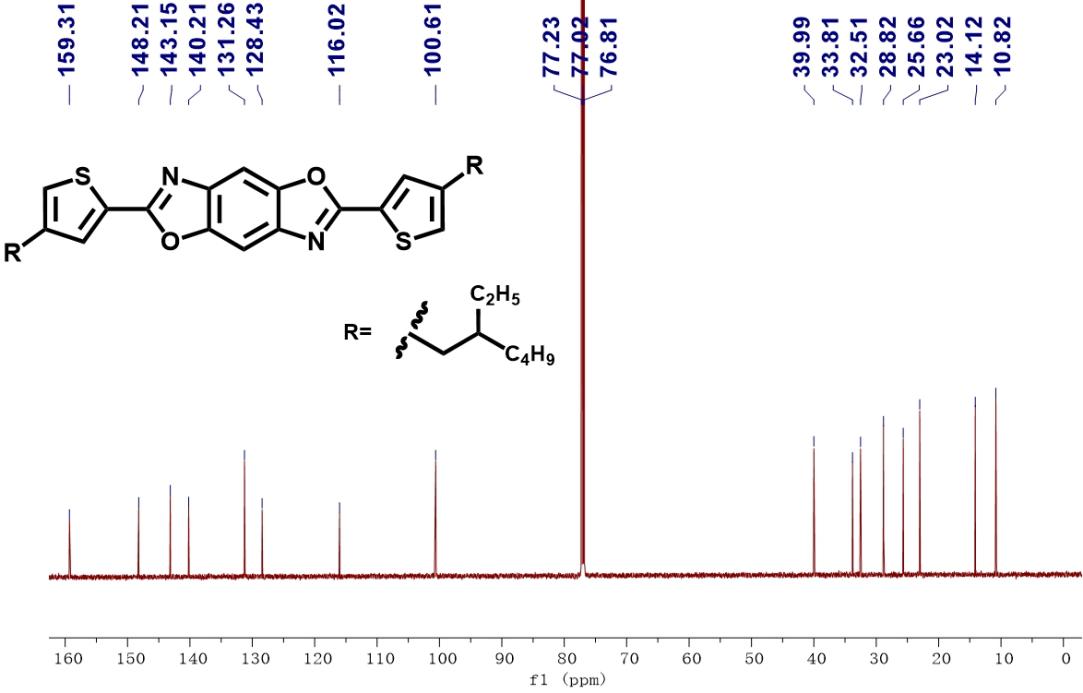


**Figure S21.** The ^13^C NMR spectrum of compound **10** in CDCl_3_ at room temperature.


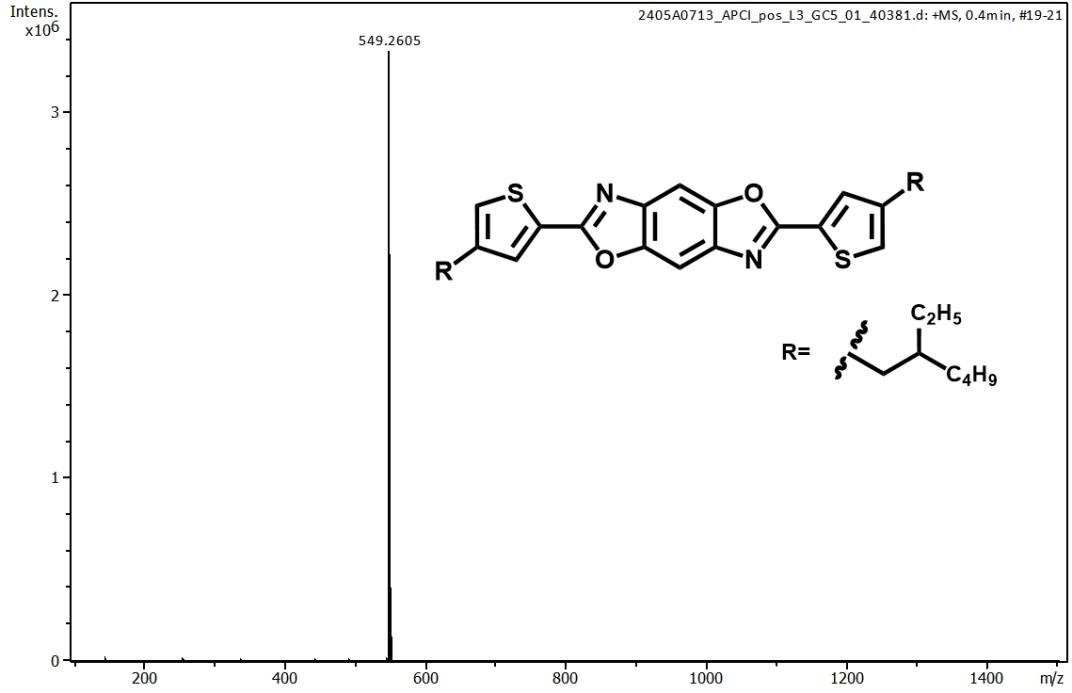


**Figure S22.** The MS spectrum of compound **10**.


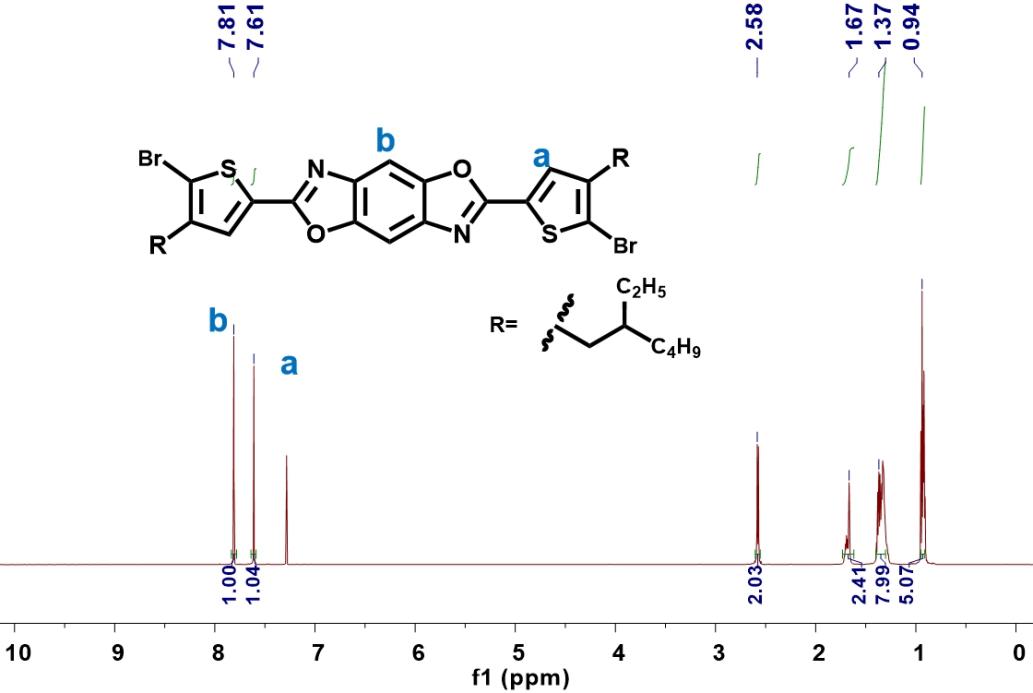


**Figure S23.** The ^1^H NMR spectrum of **M2** in CDCl_3_ at room temperature.


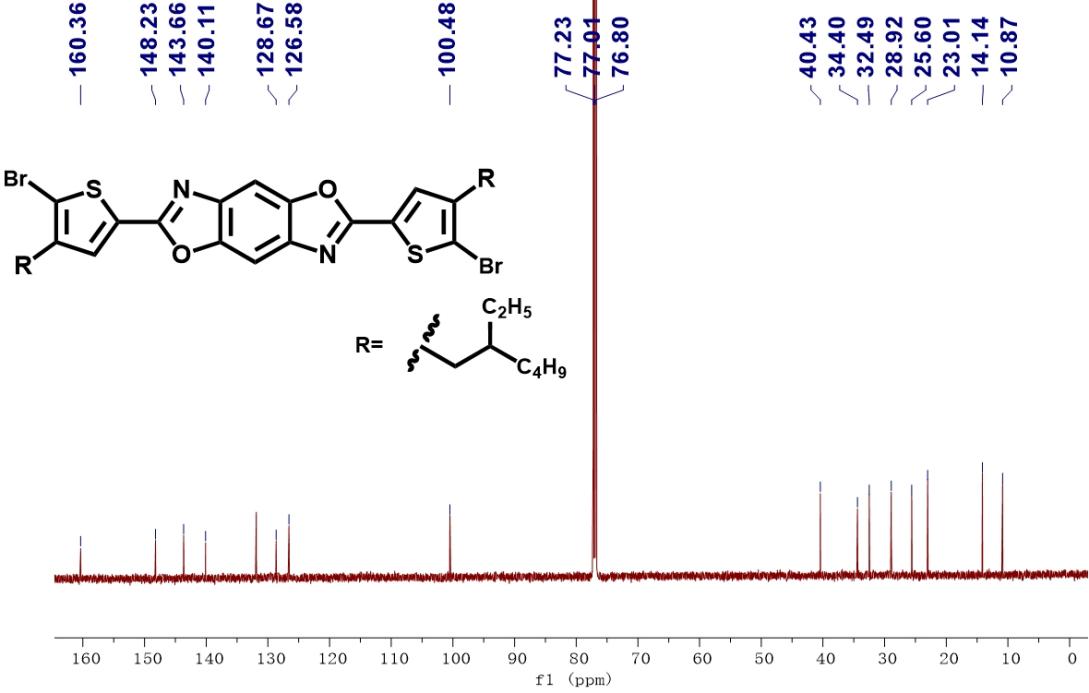


**Figure S24.** The ^13^C NMR spectrum of **M2** in CDCl_3_ at room temperature.


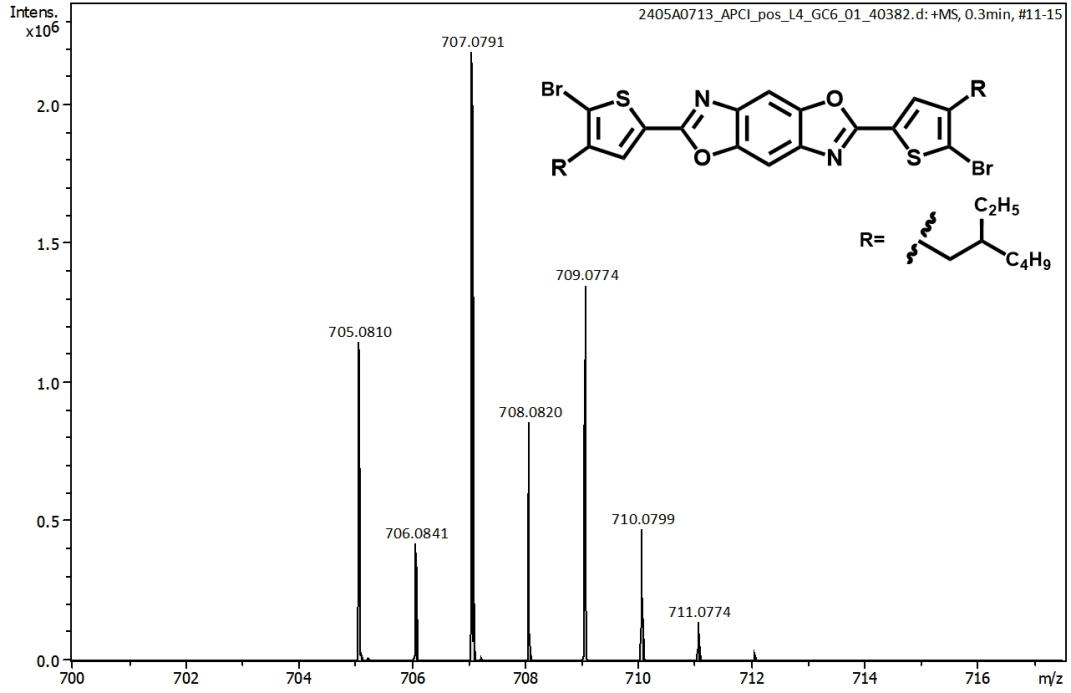


**Figure S25.** The MS spectrum of **M2**.


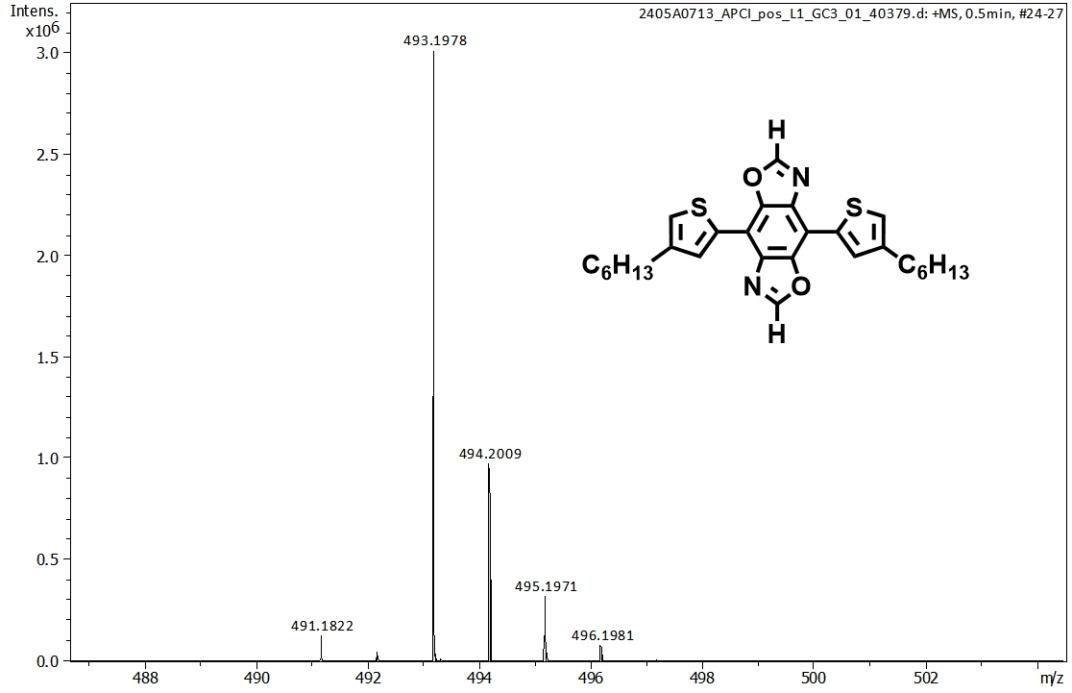


**Figure S26.** The MS spectrum of **[4,8]BBO-T**.


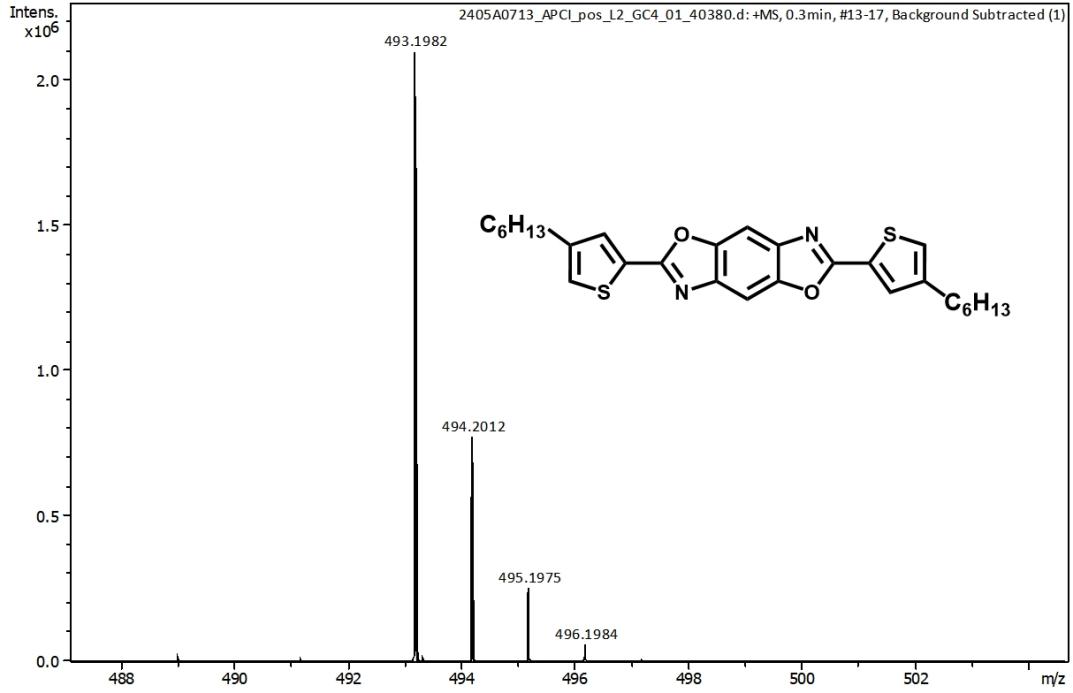


**Figure S27.** The MS spectrum of **[2,6]BBO-T**.


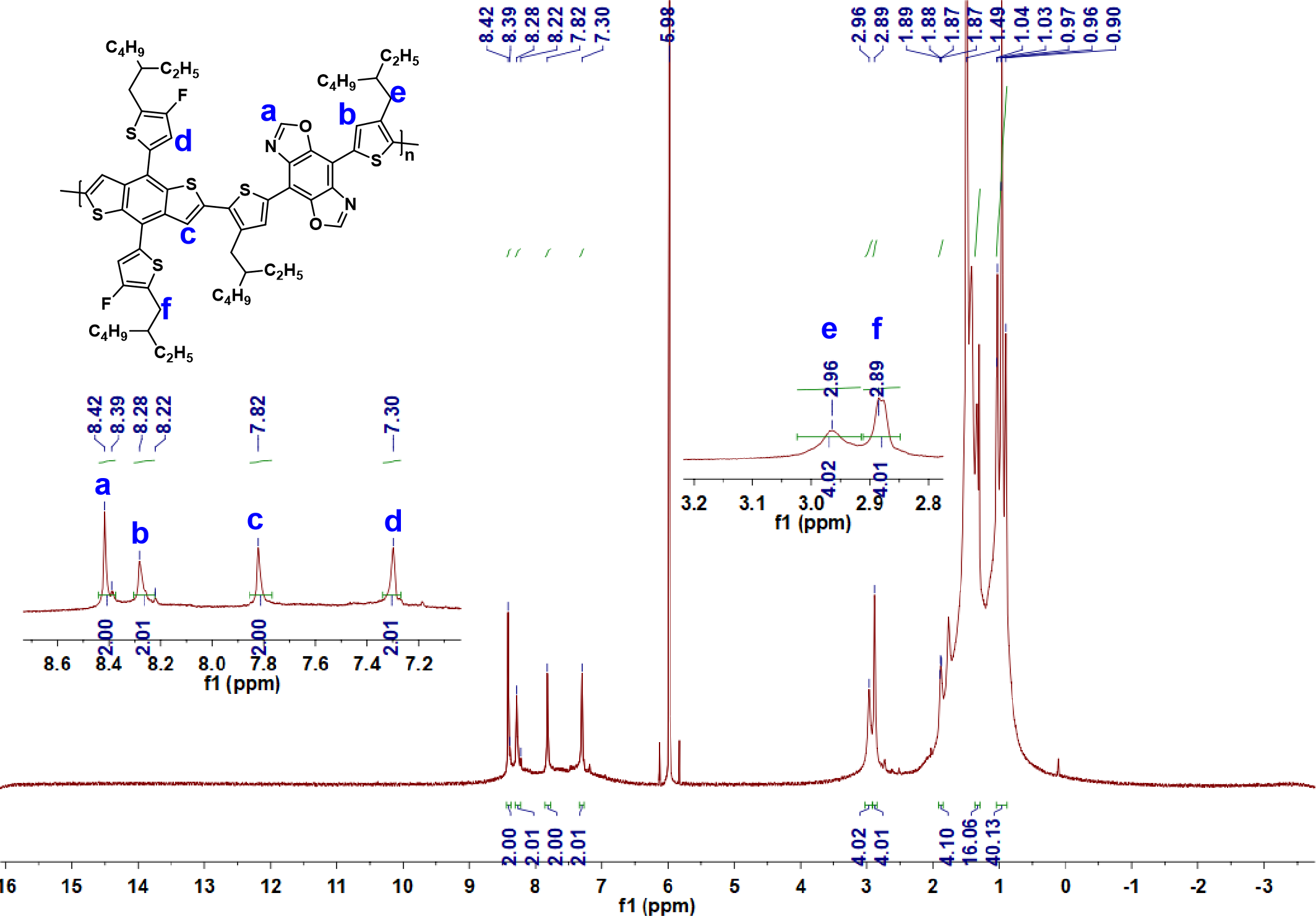


**Figure S28**. The high-temperature ^1^H NMR spectra of polymer P[4,8]BBO in C_2_D_2_Cl_4_ at 80 ^o^C.


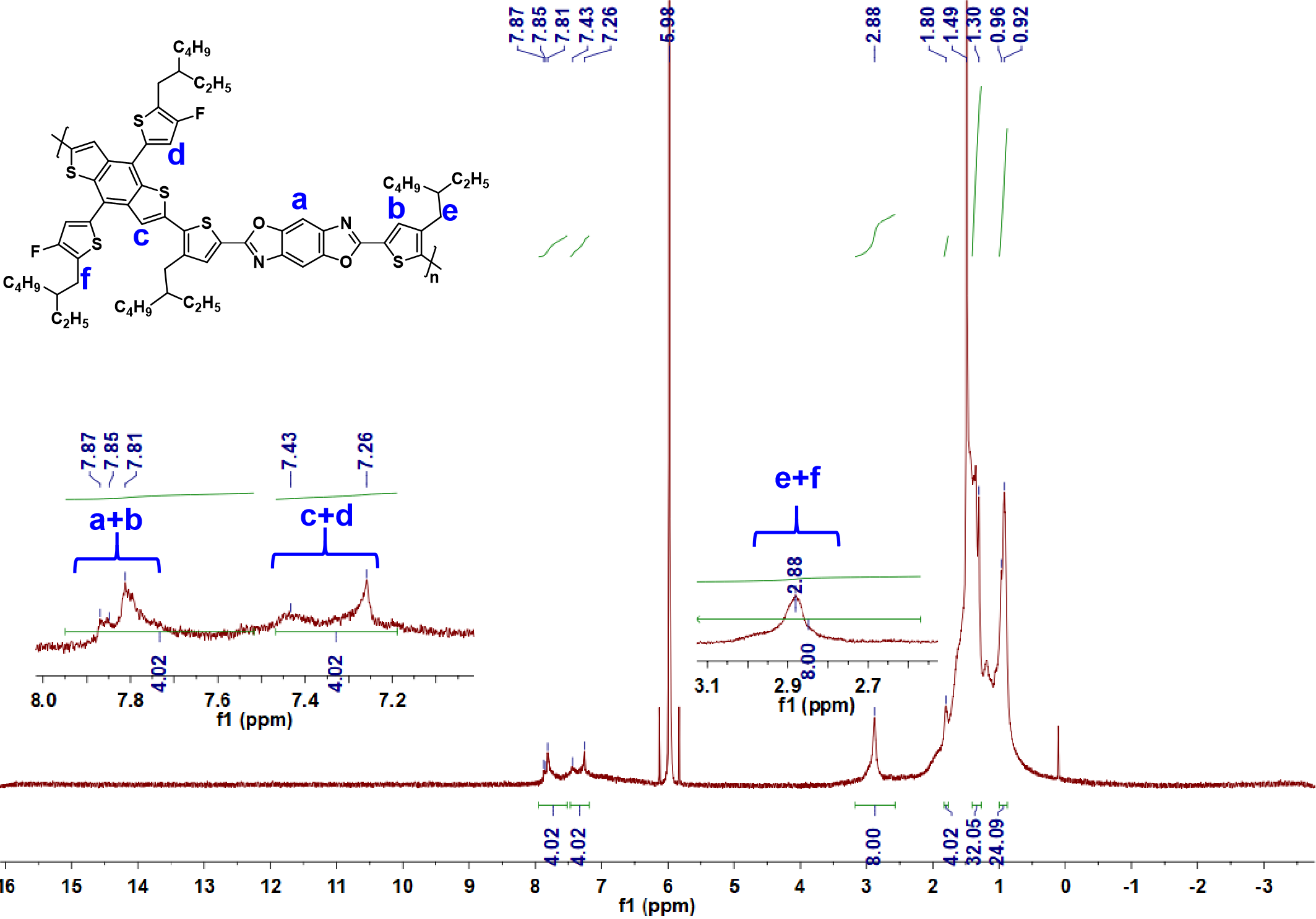


**Figure S29**. The high-temperature ^1^H NMR spectra of polymer P[2,6]BBO in C_2_D_2_Cl_4_ at 80 ^o^C.

# 15. Crystal Growth and Single Crystal Determination

X-ray data collection, structure solution, and refinement: the single crystals of [4,8]BBO-T, and [2,6]BBO-T were grown at room temperature using solvent diffusion for [2,6]BBO-T and slow evaporation for [4,8]BBO-T (dichloromethane/methanol = 9:1 in volume) in NMR tube. On the Bruker D8 Venture instrument, the single-crystal X-ray diffraction (SXRD) data of [4,8]BBO-T, and [2,6]BBO-T were collected with a Cu Kα X-ray source (λ = 1.54178 Å) at 300 K. As shown in Figure S28, the crystal information is summarized.

#
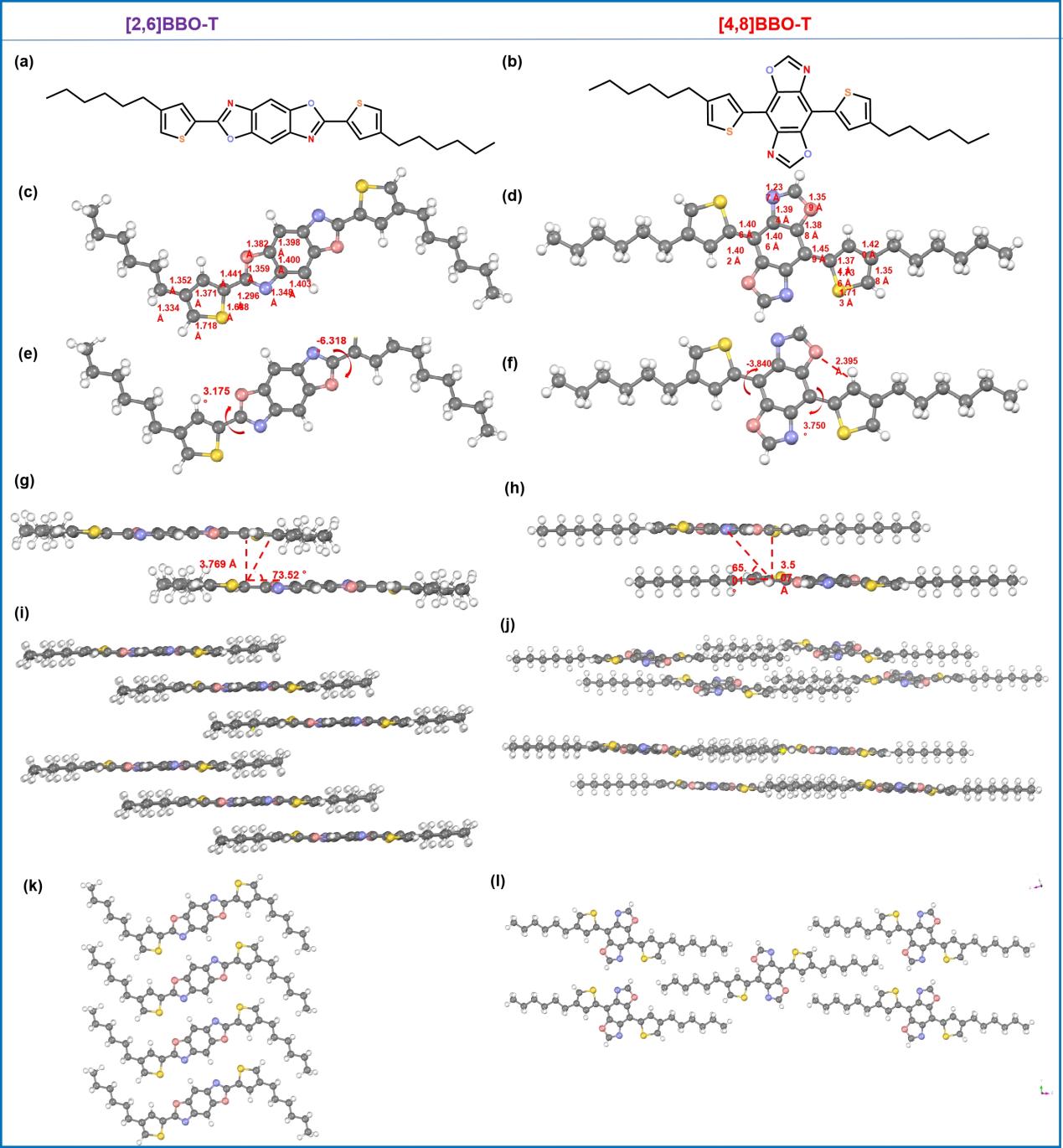


**Figure S30.** The single crystal information of model compounds.

**Table S11**. The crystal information and experimental details for [2,6]BBO-T and [4,8]BBO-T

| Compound | [2,6]BBO-T | [4,8]BBO-T |
| --- | --- | --- |
| Formula | C_28_H_32_N_2_O_2_S_2_ | C_28_H_32_N_2_O_2_S_2_ |
| Formula weight | 492.70 | 492.70 |
| Temperature/K | 296 | 240 |
| Radiation | Cu Kα (λ = 1.54184) | Cu Kα (λ = 1.54184) |
| *D_calc._* / g cm ^-3^ | 1.099 | 1.347 |
| *µ*/mm ^-1^ | 0.630 | 2.212 |
| Space Group | P -1 | P 21/n |
| *a*/Å | 6.7864 (4) | 10.8192(13) |
| *b*/Å | 9.8635 (6) | 9.2821(10) |
| *c*/Å | 11.3180 (8) | 12.2196(13) |
| *α*/° | 107.337 | 90 |
| *β*/° | 105.371 | 98.246 |
| *γ*/° | 105.327 | 90 |
| V/Å^3^ | 646.99(8) | 1214.5(2) |
| *Z* | 1 | 2 |
| *F_000_* | 214.0 | 524.0 |
| *F_000’_* | 214.73 | 526.60 |
| M_r_ | 428.30 | 493.68 |
| h | 8 | 12 |
| k | 12 | 11 |
| l | 14 | 14 |
| Theta (max) | 72.792 | 67.05 |
| T_min_ | 0.570 | 0.923 |
| T_max_ | 0.754 | 0.957 |
| N_ref_ | 2569 | 2161 |
| *w*R_2_ (all data) | 0.6079 (2437) | 0.1442 (2161) |
| R_1_ (all data) | 0.2465 (1928) | 0.0508 (1930) |

|  |
| --- |

# 16. References

[1] Gaussian 16, Revision C.02, M. J. Frisch, G. W. Trucks, H. B. Schlegel, G. E. Scuseria, M. A. Robb, J. R. Cheeseman, G. Scalmani, V. Barone, G. A. Petersson, H. Nakatsuji, X. Li, M. Caricato, A. V. Marenich, J. Bloino, B. G. Janesko, R. Gomperts, B. Mennucci, H. P. Hratchian, J. V. Ortiz, A. F. Izmaylov, J. L. Sonnenberg, D. Williams-Young, F. Ding, F. Lipparini, F. Egidi, J. Goings, B. Peng, A. Petrone, T. Henderson, D. Ranasinghe, V. G. Zakrzewski, J. Gao, N. Rega, G. Zheng, W. Liang, M. Hada, M. Ehara, K. Toyota, R. Fukuda, J. Hasegawa, M. Ishida, T. Nakajima, Y. Honda, O. Kitao, H. Nakai, T. Vreven, K. Throssell, J. A. Montgomery, Jr., J. E. Peralta, F. Ogliaro, M. J. Bearpark, J. J. Heyd, E. N. Brothers, K. N. Kudin, V. N. Staroverov, T. A. Keith, R. Kobayashi, J. Normand, K. Raghavachari, A. P. Rendell, J. C. Burant, S. S. Iyengar, J. Tomasi, M. Cossi, J. M. Millam, M. Klene, C. Adamo, R. Cammi, J. W. Ochterski, R. L. Martin, K. Morokuma, O. Farkas, J. B. Foresman, and D. J. Fox, Gaussian, Inc., Wallingford CT, 2019. D. J. Fox, Gaussian 16, Revision C.02, Gaussian, Inc., Wallingford CT, (2019).

[2] A. D. Becke, *J. Chem. Phys.* **1993**, *98*, 1372.

1. S. Grimme, J. Antony, S. Ehrlich, H. Krieg, *J. Chem. Phys.* **2010**, *132*.
2. B. P. Pritchard, D. Altarawy, B. Didier, T. D. Gibson, T. L. Windus, *J. Chem. Inf. Model.* **2019**, *59*, 4814.

[5] T. Lu, F. Chen, *J. Comput. Chem.* **2012**, *33*, 580.

[6] W. Humphrey, A. Dalke, K. Schulten, *J. Mol. Graph.* **1996**, *14*, 33.
